# Isotopic evidence of dissolved organic nitrogen production and consumption in ocean surface waters

**DOI:** 10.1073/pnas.2530315123

**Published:** 2026-10-28

**Authors:** Yeongjun Ryu, Dario Marconi, Zhou Liang, Angela N. Knapp, François Fripiat, Sarah E. Fawcett, Patrick A. Rafter, Sergey Oleynik, Alfredo Martínez-García, Gerald H. Haug, Daniel M. Sigman

**Affiliations:** ^a^ Department of Geosciences, Princeton University, Princeton, NJ 08544; ^b^ Climate Geochemistry Department, Max Planck Institute for Chemistry, Mainz 55128, Germany; ^c^ Division of Biosphere Sciences and Engineering, Carnegie Institution for Science, Pasadena, CA 91125; ^d^ Oceanography Department, Texas A&M University, College Station, TX 77843; ^e^ Department of Geosciences, Environment and Society, Université Libre de Bruxelles, Brussels 1050, Belgium; ^f^ Department of Oceanography, University of Cape Town, Rondebosch 7700, South Africa; ^g^ Marine and Antarctic Research Centre for Innovation and Sustainability, University of Cape Town, Rondebosch 7700, South Africa; ^h^College of Marine Science, University of South Florida, St. Petersburg, FL 33701; ^i^ Department of Earth and Planetary Sciences, ETH Zürich, Zürich 8092, Switzerland

**Keywords:** dissolved organic nitrogen, nitrogen cycle, isotope

## Abstract

Unlike dissolved inorganic nitrogen, which is scarce in much of the tropical, subtropical, and temperate surface ocean, dissolved organic nitrogen (DON) has significant concentrations and gradients. Accordingly, the lateral transport of DON may fuel biological productivity in nutrient-depleted low-latitude surface waters. Here, we report measurements of the concentration and ^15^N/^14^N ratio (δ^15^N) of DON in surface waters over the Pacific and Atlantic basins. DON concentration is generally highest in subpolar and equatorial waters, with minima in the subtropical gyres. A δ^15^N correlation is observed between surface DON and the nitrate consumed in the surface waters, indicating in situ DON production and consumption. Samples with lower DON concentrations tend to have anomalously high DON δ^15^N, confirming net DON transport to, and consumption in, the subtropical gyres. Net local DON production or consumption appears to dominate the observed spatial variation in the DON-to-subsurface nitrate δ^15^N difference. For example, N_2_ fixation tends to lower the δ^15^N of surface DON relative to subsurface nitrate mostly through encouraging DON accumulation rather than by the specific δ^15^N of the nitrogen introduced by N_2_ fixation. Unique conditions appear to apply in the equatorial Atlantic, where despite high productivity, a local minimum in DON concentration and a slight elevation in DON δ^15^N suggest net DON degradation. In this phosphorus-rich, nitrogen-poor upwelling environment where low iron may restrict N_2_ fixation, net decomposition of DON may supply needed recycled nitrogen to the ecosystem.

## Significance

Dissolved organic nitrogen (DON) is an abundant yet poorly understood nitrogen form in the surface ocean. We report a basin-scale dataset of DON concentrations and nitrogen isotopes from the Pacific and Atlantic Oceans. The nitrogen isotopes confirm in situ production and consumption of surface DON and show that its lateral transport redistributes nitrogen from high-productivity regions to nutrient-depleted subtropical gyres. Additionally, in the equatorial Atlantic, where phosphorus is available but low iron limits N_2_ fixation, DON degradation may supply recycled nitrogen as an alternative to newly fixed nitrogen. For the least productive low-latitude surface ocean regions, where circulation and density stratification impede nutrient supply from below, our results indicate that dissolved organic matter transport in surface waters augments biological production.

Autotrophic microbial productivity in the tropical and subtropical ocean is often limited by nitrogen (N) availability, with these low latitude waters exhibiting extremely low concentrations of dissolved inorganic N (DIN), including its most important forms, nitrate and ammonium (1). Yet, surface waters bear substantial concentrations (>3 µM) of dissolved organic nitrogen (DON) (2, 3), suggesting the presence of a large organic N reservoir despite DIN scarcity.

The persistence of DON in surface waters initially led to the view that much of the DON pool is biologically inert. However, subsequent studies revealed spatial gradients in DON concentrations across the surface ocean (3–5), implying active production and consumption. Such findings raise questions about the origin of DON, what controls its mean concentration and variation, and whether it is an important N source to upper ocean ecosystems. These questions have motivated efforts to constrain the biogeochemical cycling of DON, but progress has been limited by its chemical complexity. Molecular characterizations of partial DON fractions isolated through chemical separations indicate that high molecular weight components are enriched in amide-containing compounds (6, 7), reflecting contributions from labile biopolymers such as amino acids and amino sugars. The low molecular weight fraction, which is proportionally most important in the deep ocean, is dominated by heterocyclic compounds that are more refractory to decomposition (8). However, these characterization studies capture only portions of the DON pool, with the composition and bioreactivity of the rest still uncertain. The chemical heterogeneity of DON also complicates the interpretation of isotope tracer addition experiments. Given these challenges, natural-abundance isotopic approaches have been pursued to investigate DON dynamics and its role in upper-ocean biogeochemical cycles (9–13).

DON is both a product of photosynthetic growth (14) and a potentially bioavailable organic nutrient (15). Upper-ocean DON concentrations often exhibit spatial gradients, with a tendency toward lower concentrations in the oligotrophic gyres (3–5). This spatial pattern, which appears to be qualitatively similar for dissolved organic phosphorus (DOP) (16, 17), may indicate regions of net production and consumption of dissolved organic nutrients (18). Biogeochemical models now regularly incorporate these dynamics, estimating that lateral transport of dissolved organic nutrients fuels a significant fraction of export production in subtropical gyres (18, 19). Lateral transport of DON has been estimated to support ~15% of subtropical gyre production (18), potentially explaining a significant part of the excess of observed export production relative to predictions for these regions (20). However, compiled DON concentration data are variable (21), and the controls on the DON cycle in the open ocean remain poorly understood. Moreover, while concentration gradients of DON suggest regions of net DON production and consumption across the ocean surface, they do not prove them: The surface DON concentration in any given region could simply represent the local balance of production and consumption, as, for example, roughly applies to euphotic zone chlorophyll concentration. Thus, more direct tracers of net production and consumption of DON are needed.

Biological N_2_ fixation by diazotrophs is another key mechanism for supplying bioavailable N to open ocean ecosystems (22), with additional inputs such as atmospheric deposition and riverine supply (23–26). In contrast to DON decomposition, which is solely an aspect of the ocean’s internal N cycling, N_2_ fixation represents the dominant ultimate source of biologically available (i.e., “fixed”) N to the ocean. The N demands of microbial communities can be fulfilled by N_2_ fixation when specific environmental conditions are satisfied. These include appropriate temperature and light conditions and at least two nutrients: phosphorus (P), a critical component of all life, and the trace metal iron, which is required in relatively large amounts by the enzyme complex of nitrogenase that catalyzes N_2_ fixation (27, 28). It has long been argued that N_2_ fixation requires available P but depletion of N, as otherwise non-N_2_-fixing phytoplankton will outcompete N_2_-fixing phytoplankton for nutrients (29, 30). However, there is not as yet consensus as to the importance of N impoverishment relative to other conditions (31, 32).

To date, research into the interaction of dissolved organic nutrients with N_2_ fixation has focused on DOP consumption and DON production. Net DOP production in upwelling systems is observed to accumulate a P reservoir in surface waters that can be consumed in downstream waters to drive N_2_ fixation even after inorganic P has been completely depleted (19, 33, 34). Conversely, it has been suggested that N_2_ fixation adds substantially to the surface DON reservoir (35), providing fixed N that can be drawn upon subsequently by the plankton of the low-nutrient surface ocean.

**Fig. 1.**
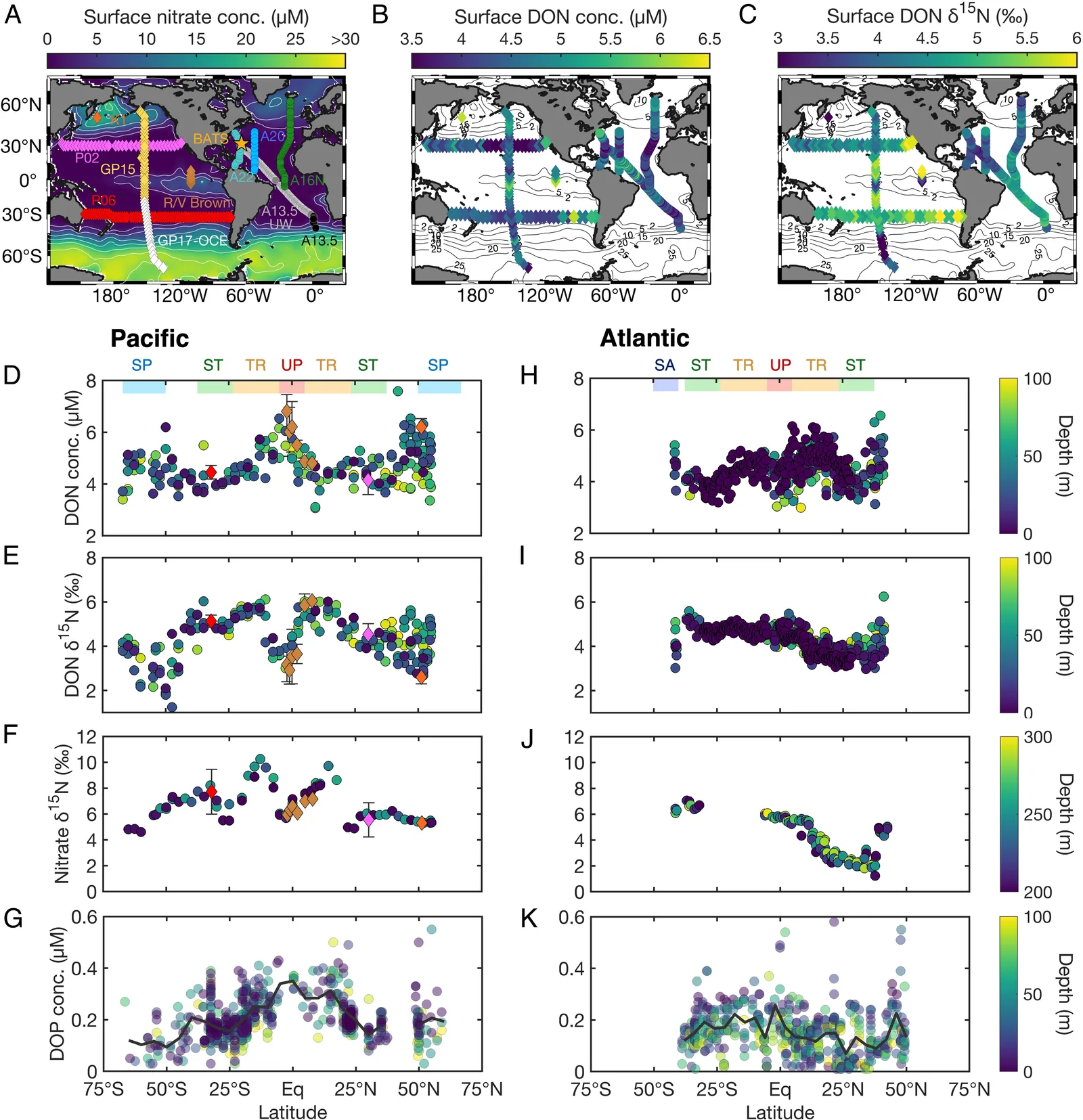
Surface ocean distribution of DON concentration and δ^15^N. (*A*) Sampling sites are indicated by symbols: yellow diamonds (GEOTRACES GP15), white diamonds (GEOTRACES GP17-OCE), pink diamonds (P02 2013), red diamonds (P06 2017), orange diamond (Station K1), brown diamonds (R/V *Brown* 2003), black circles (A13.5 2020), gray circles (A13.5 2020 underway stations without depth-resolved samples), green circles (A16N 2013), cyan circles (A22 2021), blue circles (A20 2021), and yellow star (BATS station 2022). Background shading and contours show annual mean surface nitrate concentrations (36). (*B*) Surface mean DON concentrations and (*C*) δ^15^N values (upper 100 m) are color-coded and overlaid with surface nitrate concentration contours (μM). (*D*–*K*) Meridional distributions of DON concentration (*D* and *H*), δ^15^N of DON (*E* and *I*), DOP (*G* and *K*) in the upper 100 m, and (*F* and *J*) subsurface nitrate δ^15^N (200 to 300 m). Panels (*D*–*G*) show the Pacific, and panels (*H*–*K*) show the Atlantic. In (*D*–*G*), DON data from Station K1, R/V *Brown*, P02, and P06 are shown as upper-100 m averages with error bars (± 1SD), plotted as diamonds color-coded by location as in (*A*). In (*H*–*K*), data north of 8.5°N are from the Sargasso Sea, as the cruise tracks diverge beyond this latitude (see *SI Appendix*, Fig. S2 for full Atlantic coverage). Data points in (*D*–*K*) are colored by sample depth (m), and oceanographic regimes are indicated by shaded bands in the *Top* panels [red: upwelling zone (UP), orange: tropics (TR), green: subtropics (ST), blue: subpolar regions (SP), dark blue: subantarctic region (SA)]. Compiled DOP data (17) are included for comparison in (*G* and *K*), with Pacific data spanning 100°W–180° and Atlantic data from the Atlantic Meridional Transect (AMT) cruises. In (*G* and *K*), black solid lines denote moving averages calculated over 4° latitude bins.

Here, we report concentrations and N isotopic ratios [δ^15^N, where δ^15^N = ((^15^N/^14^N)_sample_/(^15^N/^14^N)_air N2_ – 1) * 1,000, in units of ‰] of DON in shallow (≤100 m) ocean waters along meridional and zonal transects through the Atlantic and Pacific basins (Fig. 1 and *SI Appendix*, Fig. S1), comparing these with previous measurements of DOP concentration and iron availability. The isotopic data provide new and independent support for the paradigm of dissolved organic nutrient transport from the nutrient-rich regions of net upwelling to the nutrient-depleted subtropical gyres, where net DON production and consumption predominantly occur, respectively. However, they also identify an apparent exception in the equatorial Atlantic that may arise from iron limitation of N_2_ fixation. These findings point to a diversity of roles that dissolved organic nutrients can play in upper ocean ecosystems.

## Results

The Atlantic samples and some of the Pacific samples were unfiltered and thus include particulate N (PN; see *Materials and Methods*). However, the PN contribution to the overall N pool is negligible (typically ≤0.3 µM) (37), such that our measurements, calculated as the difference between total dissolved N (TDN) and DIN (*Materials and Methods*), primarily reflect DON and are hereafter referred to as such. The one exception is the subpolar North Atlantic (50°–66.5°N), where samples may have higher PN concentrations, up to 1 µM (38).

Focusing first on the Pacific, DON concentrations in the upper 100 m across the Pacific basin show distinct regional patterns, with maxima in the equatorial upwelling zone (5°S–5°N; 5.6 ± 0.7 µM, mean ± 1SD) and elevated concentrations in subpolar regions (50°–66.5°N: 5.2 ± 0.6 µM and 50°–66.5°S: 4.7 ± 0.4 µM) (Fig. 1 *B* and *D*). These regions have substantial unused nitrate (>2 µM) in surface waters (Fig. 1*A*). Within the subpolar regions, strong vertical and spatial variability in DON concentrations is observed. In addition, the zonal transects across the subtropical North and South Pacific indicate elevated DON concentrations along the eastern margins (5.5 ± 0.2 µM) (*SI Appendix*, Fig. S1). In contrast, the subtropical gyres (23.5°–35°N and S) of both the North and South Pacific, where surface nitrate is fully depleted, exhibit lower DON concentrations, averaging 4.5 ± 0.3 µM and 4.1 ± 0.2 µM, respectively.

Turning to the Atlantic, elevated DON concentrations are observed in the subantarctic South Atlantic (4.6 ± 0.2 µM), the temperate North Atlantic over the eastern North American margin (5.2 ± 0.4 µM), and the subpolar North Atlantic (4.9 ± 0.3 µM), while the subtropical gyres of both the North and South Atlantic exhibit minima (4.1 ± 0.4 µM and 3.8 ± 0.3 µM, respectively) (Fig. 1 *B* and *H* and *SI Appendix*, Fig. S2). Whereas the equatorial upwelling zone of the Pacific (at ~5°S) shows a local maximum (5.9 ± 0.8 µM), the equatorial Atlantic (5°S–5°N) hosts a local minimum in surface DON concentration (4.3 ± 0.3 µM). Moreover, in contrast to the pattern observed in the Pacific, the Atlantic DON concentrations appear to have a hemispheric asymmetry, with high DON in the northern tropics (5°–23.5°N, 4.8 ± 0.6 µM). In both basins, the meridional distribution of our DON concentration measurements is generally consistent with previous observations (21), while providing a clearer picture than that arising from data compilation (*SI Appendix*, Fig. S3).

The δ^15^N of surface DON in the Pacific reveals notable spatial patterns (Fig. 1 *C* and *E*). Low δ^15^N values (station means of 1.9−2.9‰ for the upper 100 m) are characteristic of high-nutrient, high-productivity waters such as the Pacific equatorial upwelling zone and the subpolar regions, corresponding to high DON concentrations. As with DON concentration, surface DON δ^15^N in the subpolar regions spans a wide range. The DON concentration maximum and δ^15^N minimum observed in the equatorial Pacific appear even more pronounced in the *R/V Brown* samples, collected further to the east of GP15 (Fig. 1 *D* and *E*). In both the subtropical North and South Pacific, DON δ^15^N exhibits a zonal trend, decreasing from the eastern margins (5.8 ± 0.3‰) toward the interior of the gyres (Fig. 1*C* and *SI Appendix*, Fig. S1). In the subtropical North Pacific gyre, δ^15^N values are on average 4.2 ± 0.3‰, lower than those in the neighboring tropical Pacific (5.3 ± 0.4‰), with the data consistent with previous findings (10, 12). Compared to the subtropical North Pacific gyre waters at 25°N, the subtropical South Pacific gyre waters at 25°S host a less prominent local δ^15^N minimum of ~4.8 ± 0.1‰.

As with DON concentration, DON δ^15^N in the Atlantic has an interhemispheric difference, with lower DON δ^15^N in the North than the South Atlantic (Fig. 1*I*). The lowest values (~3.2‰) occur in the Sargasso Sea (i.e., the western subtropical North Atlantic), while the subtropical South Atlantic lacks such low δ^15^N values. Instead, surface DON δ^15^N is between 4.3‰ and 5.1‰ across the subtropical-to-tropical South Atlantic. From values of up to ~5.1‰ near the equator, DON δ^15^N declines into the tropical North Atlantic toward the Sargasso Sea before rising again toward the eastern North American margin and subpolar North Atlantic. In the eastern subtropical North Atlantic, DON δ^15^N also reaches relatively low values (~3.7‰), approaching those in the Sargasso Sea, consistent with previous observations (39). The low δ^15^N signal in DON occurs further north in the eastern than the western North Atlantic and appears to span a narrower meridional range (Fig. 1*C* and *SI Appendix*, Fig. S2). These west-to-east differences are consistent with the zonal asymmetry of the subtropical gyre, including its maximum thermocline depth toward the western side. Stepping back to take a broader view, the Pacific also has a lower δ^15^N of subtropical gyre DON in the northern hemisphere than in the southern hemisphere, but the interhemispheric difference is weaker than in the Atlantic. Further north in the eastern Atlantic (i.e., A16N), DON concentration increases while DON δ^15^N remains low.

**Fig. 2.**
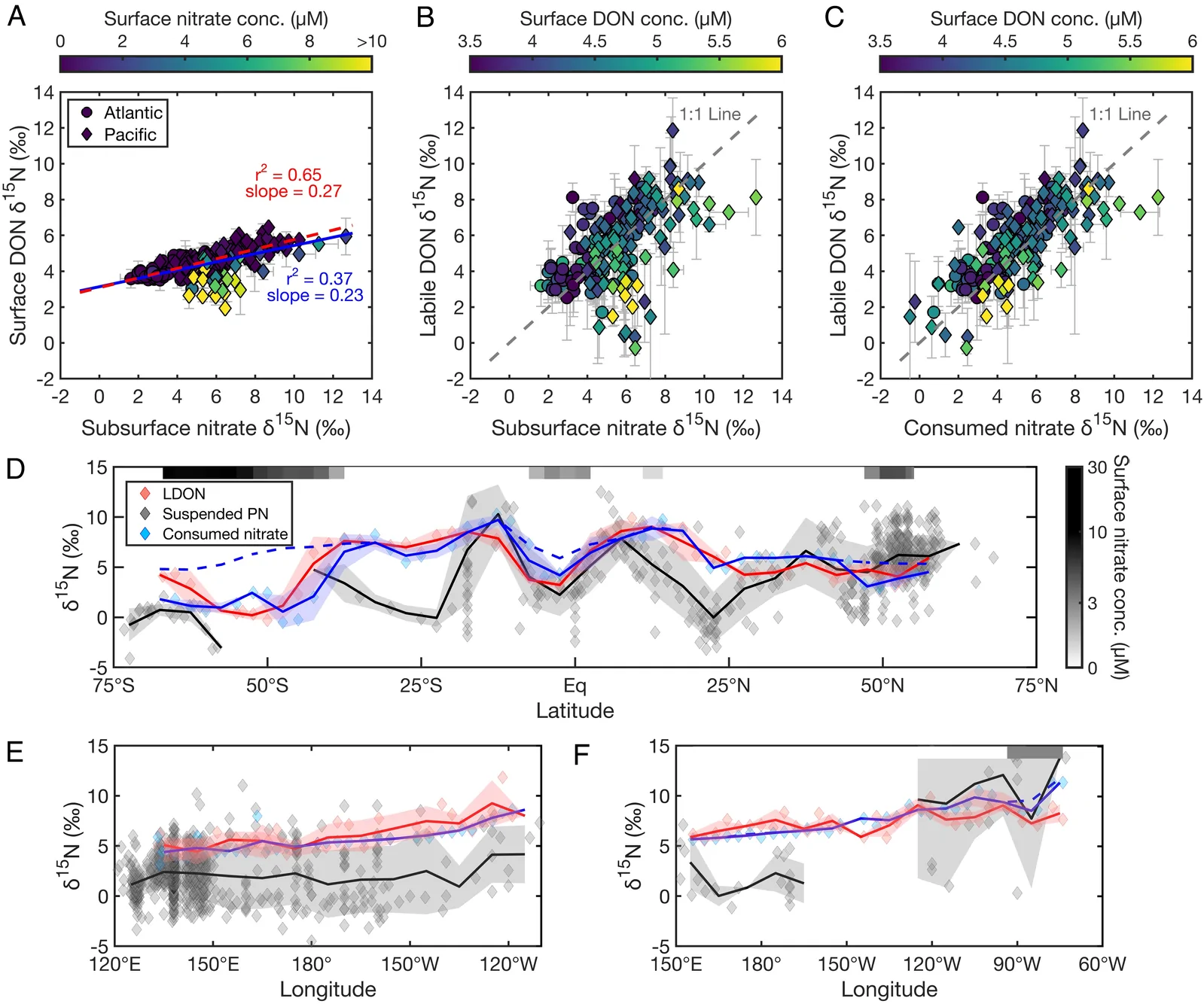
Surface DON δ^15^N in relation to subsurface nitrate and surface suspended particulate nitrogen (PN) δ^15^N across the Atlantic and Pacific basins. (*A*) Surface DON δ^15^N *vs.* subsurface nitrate δ^15^N. (*B*) Labile DON δ^15^N *vs.* subsurface nitrate δ^15^N. (*C*) Labile DON δ^15^N *vs.* the δ^15^N of the nitrate consumed in the upper 100 m. (*D*) Meridional distributions of δ^15^N of labile DON (red) and consumed nitrate (blue), and the δ^15^N of suspended PN from the upper 100 m (gray) along Pacific transects near 150°W (GP15 and GP17-OCE). Suspended PN δ^15^N data are shown for the 130°–170°W longitude range (40). (*E* and *F*) Zonal distributions of the same parameters along the subtropical North Pacific (P02, ~30°N) and subtropical South Pacific (P06, ~30°S) transects, respectively. Suspended PN δ^15^N data are shown for 20°–40°N in (*E*) and 20°–40°S in (*F*) (40). In (*A*–*C*), surface DON and labile DON δ^15^N values are averages for the upper 100 m, whereas consumed nitrate δ^15^N values are calculated as δ^15^N_consumed_ = (δ^15^N_subsurface_*[NO_3_^−^]_subsurface_ – δ^15^N_surface_*[NO_3_^−^]_surface_)/([NO_3_^−^]_subsurface_ – [NO_3_^−^]_surface_), where the subscripts “surface” and “subsurface” represent the mean values for the upper 100 m and the 200 to 300 m depth intervals, respectively. Error bars indicate ± 1SD of measurements within the corresponding depth intervals; for labile DON δ^15^N, the SD was propagated through the mass balance. Circles denote Atlantic samples and diamonds denote Pacific samples. Data points are colored according to surface nitrate concentration in (*A*) and surface DON concentration in (*B* and *C*). The blue solid line in (*A*) indicates the linear regression for all samples, whereas the red dashed line indicates the regression for samples with surface nitrate concentrations <1 μM. Gray dashed lines in (*B* and *C*) indicate a 1:1 reference line rather than a regression fit. In (*D*–*F*), red and blue symbols denote depth-averaged labile DON δ^15^N (upper 100 m) and consumed nitrate δ^15^N, respectively, whereas gray symbols represent individual suspended PN δ^15^N measurements (40). Gray-scale bars along the top indicate surface nitrate concentrations. Solid red, blue, and gray lines show latitudinal or longitudinal bin means of labile DON δ^15^N, consumed nitrate δ^15^N, and suspended PN δ^15^N, respectively; shaded envelopes indicate ± 1SD (5° latitudinal bins in *D*; 10° longitudinal bins in *E* and *F*). Blue dashed lines indicate mean subsurface nitrate δ^15^N within each latitude or longitude bin. Because a common y-axis scale is used across panels *D*–*F*, a small number of suspended PN δ^15^N values fall outside the plotted ranges.

Overall, the spatial patterns in the δ^15^N of surface DON (Fig. 1 *E* and *I*) follow those of subsurface nitrate δ^15^N (Fig. 1 *F* and *J*). The δ^15^N of surface DON is positively correlated with the δ^15^N of subsurface nitrate, i.e., mean nitrate δ^15^N at 200 to 300 m, a depth range chosen to avoid the isotopic signal of assimilation by phytoplankton and thus characterize the δ^15^N of vertical nitrate supply to the euphotic zone (Fig. 2*A*). The correlation is stronger if considering only samples from stations with surface nitrate concentrations of less than 1 µM (Fig. 2*A*). However, the range of surface DON δ^15^N variation is significantly smaller than that of subsurface nitrate, with only about one fourth of the δ^15^N variation that is observed in nitrate, yielding a linear regression slope of 0.23 to 0.27 (Fig. 2*A*).

## Discussion

### Evidence for DON Pools With Different Cycling Rates.

The observed meridional trend in DON concentration generally aligns with previous studies (2, 3, 21), with higher concentrations in high-productivity regions and lower concentrations in low-productivity areas like the subtropical gyres (*SI Appendix*, Fig. S3). Our highest DON concentrations are observed in the equatorial Pacific upwelling zone and in subpolar regions, where nitrate is available in the surface waters (Fig. 1*B*). This finding supports the view that in regions with replete inorganic N (i.e., nitrate), the production of DON exceeds its degradation, leading to DON accumulation (12). In contrast, the low DON concentrations of the subtropical gyres suggest net DON degradation, likely driven by the nutrient impoverishment of these regions. The exceptional cases of the equatorial Atlantic and tropical North Atlantic, with low surface DON in the former and relatively high DON in the latter, are discussed further below.

We must note a caveat with regard to seasonality. In polar regions, seasonality in biological productivity, combined with rapid surface-subsurface exchange during the nonsummer seasons, can dilute the DON being produced in the surface, lowering DON concentration while tending to raise DON δ^15^N in the surface ocean. Indeed, these processes may explain the relatively high variability in the subpolar North Pacific (Fig. 1 *D* and *E*). Such seasonal fluctuations are minimal in the low-latitude ocean, with observations from the Sargasso Sea suggesting that DON concentrations and δ^15^N values are generally stable throughout the year (9). The correlation in δ^15^N between surface DON and subsurface nitrate (Fig. 2*A*) suggests that new DON production fueled by the local subsurface nitrate supply contributes a portion of the surface DON pool (10, 41). Without overlapping rapid production and consumption, the transport of DON from one region to the next with different subsurface nitrate δ^15^N would destroy the δ^15^N correlation between subsurface nitrate and surface DON. Thus, given the rate of wind-driven upper ocean circulation, achieving this correlation requires production and decomposition of the reactive component of the DON in surface waters on timescales of months up to a year (41, 42). However, the slope of ~0.25 for the isotopic correlation between surface DON and subsurface nitrate (Fig. 2*A*) suggests that this reactive DON is only ~25% of the total DON pool (i.e., ~1 µM) in nitrate-poor surface waters. That is, the low correlation slope suggests that most surface DON is unaffected by regional production, consistent with the persistence of a “nonlabile” DON pool (43) that is produced and consumed on much longer time scales and, thus, is more uniform across the ocean. Most likely, there is a spectrum of reactivity in DON, such that the most accurate model would include multiple DON pools undergoing various rates of production, consumption, and interconversion (44). Here, for simplicity, we assume a two-pool model (45), with the nonlabile pool assumed to be uniform in concentration and δ^15^N. For the nonlabile DON pool, deep-water (≥1,000 m) measurements from the Bermuda Atlantic Time-series Study (BATS) station were used, yielding an estimated concentration and δ^15^N of 2.9 ± 0.4 µM and 3.9 ± 0.4‰, respectively (*SI Appendix*, Fig. S4; see *SI Appendix*).

With subtraction of this nonlabile DON pool, we find that the δ^15^N of the reactive DON fraction (hereafter, labile DON) exhibits a higher slope of correlation with the δ^15^N of subsurface nitrate (Fig. 2*B*; 1:1 reference line). However, in surface waters where nitrate is not completely exhausted, the δ^15^N of labile DON is consistently lower relative to its subsurface nitrate source. This low δ^15^N in labile DON from nitrate-rich regions can be at least partly attributed to isotopic fractionation during nitrate assimilation by phytoplankton: Due to the preferential uptake of the lighter nitrogen isotope during partial nitrate assimilation (46, 47), the newly produced biomass N possesses a lower δ^15^N value than the initially supplied nitrate. To account for incomplete nitrate assimilation, we estimated the δ^15^N of the consumed nitrate in the surface layer, assuming that the nitrate drawdown from the subsurface to the surface is driven by biological uptake (*SI Appendix*). Comparing the consumed nitrate δ^15^N with labile DON δ^15^N improves the agreement with 1:1 correlation (Fig. 2*C*).

The ~1:1 covariation of labile DON δ^15^N with consumed nitrate δ^15^N implies that the production of this labile DON pool is dominantly fueled by the regional nitrate supply. Yet, despite this overall alignment, plotting labile DON δ^15^N against consumed nitrate δ^15^N reveals distinct deviations associated with DON concentrations, with coherent sets of samples falling systematically above or below the 1:1 line (Fig. 2*C*). As indicated by the coloring, many of the samples above the line have lower DON concentrations, while many below have higher DON concentrations. Consequently, the isotopic offset between labile DON and consumed nitrate (denoted as LDON-NO__3_^−^_ Δδ^15^N, equal to labile DON δ^15^N – consumed nitrate δ^15^N) exhibits a negative correlation with DON concentration (*SI Appendix*, Fig. S5). The sense of the deviation, with the δ^15^N of labile DON falling above or below the 1:1 line when the DON concentration is lower or higher, points to regions of net consumption and production of surface DON, respectively, based on the following arguments.

**Fig. 3.**
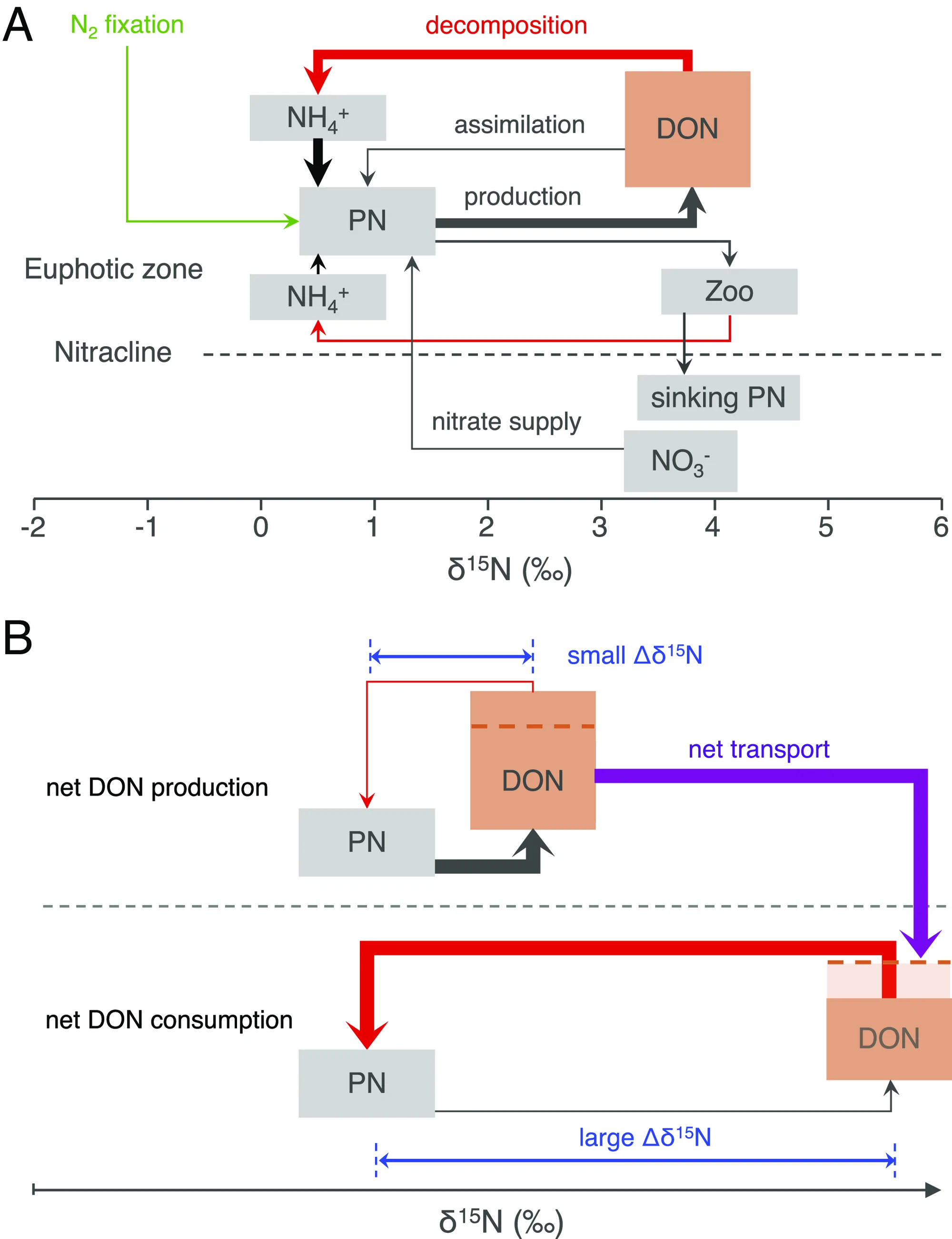
Conceptual schematics for DON δ^15^N. (*A*) Schematic illustrating the controls on DON δ^15^N under balanced DON production and consumption, modified from an oligotrophic case (10). (*B*) Deviation of DON δ^15^N from suspended PN δ^15^N under net DON production (*Upper*) and net DON consumption (*Lower*), relative to the balanced case (dashed box). In (*A*), the upper loop represents the microbial loop associated with DON production and decomposition, whereas the lower loop denotes zooplankton metabolism. Solid black arrows indicate processes assumed to occur without isotopic fractionation or for which isotopic fractionation is not expressed (e.g., with full consumption), while red arrows highlight processes with isotopic fractionations that are consequential for DON. The green solid arrow represents the contribution of N_2_ fixation (δ^15^N ≈ −1‰) (35). For simplicity, DON refers only to the labile fraction, and complete nitrate consumption is considered, making the consumption a nonfractionating process; otherwise, due to the nitrate assimilation isotope effect, incomplete nitrate consumption lowers the δ^15^N of the consumed nitrate, in turn, lowering the δ^15^N values of the other N pools in the euphotic zone. Arrow thickness reflects the relative intensity of each process. In (*B*), the resulting δ^15^N offset between suspended PN and labile DON (Δδ^15^N, in blue) is determined by the balance between DON production and degradation and is illustrated by the length of the blue arrow in each case. The purple arrow indicates net transport of DON from the production region to the consumption region. The heights of the DON boxes indicate DON concentration, with the orange dashed line referring to the DON concentration in the balanced case of (*A*).

DON is produced through multiple pathways linked to PN, including direct exudation from phytoplankton, release of newly fixed N by diazotrophs, grazing and sloppy feeding by zooplankton, and the solubilization or microbial decomposition of PN (48, 49). The production of PN and then DON is dominantly fueled by nitrate supplied to the euphotic zone (3). The PN in surface waters (i.e., suspended PN) typically has a low δ^15^N. Across most of the surface ocean, this is dominantly due to heterotrophs’ preferential metabolic recycling and release of low-δ^15^N ammonium that is then reassimilated by the phytoplankton in the suspended PN pool, while higher-δ^15^N heterotroph N is preferentially exported by the sinking flux (37). In nitrate-bearing surface waters, isotopic fractionation during incomplete nitrate assimilation also produces PN with a low δ^15^N (47). Finally, in N-poor settings, an additional source of N to the euphotic zone is N_2_ fixation (50–52), with a low δ^15^N of ~–1‰ (35), which probably most frequently appears first as PN. Freshly produced DON is expected to inherit a δ^15^N similar to suspended PN and, thus, lower than that of the nitrate supply (Fig. 3*A*). Microbial consumption tends to preferentially remineralize low-δ^15^N DON (10), producing ammonium that is rapidly assimilated into the suspended PN pool. Some phytoplankton can directly assimilate DON (53), which may not have an isotopic effect (e.g., ref. 46). However, DON remineralization is likely to represent the dominant pathway of DON turnover, supported by negative correlation observed between DON concentration and δ^15^N (12, 13). Consequently, microbial consumption of DON raises the δ^15^N of the DON relative to its PN sources (Fig. 3*A*).

Together with DON production, the isotopic fractionation during degradation provides a framework for interpreting the observed variations in DON δ^15^N (Fig. 3*B*). Over an adequately large region of the ocean surface, one can assume a steady-state balance between labile DON production and consumption (10) (Fig. 3*A*). Under this steady state, the δ^15^N of DON is expected to be elevated relative to the δ^15^N of newly produced DON (and, thus, of PN) by the isotope effect associated with DON degradation. Despite the sparse availability of suspended PN δ^15^N data, comparison of our estimates of labile DON δ^15^N with suspended PN δ^15^N across the Pacific indicates that labile DON is on average ~1.8‰ higher (Fig. 2 *D*–*F*), implying that DON remineralization occurs with a normal isotope effect (i.e., preferential remineralization of low-δ^15^N DON) (12, 13). In regions of net DON production, the labile DON-PN δ^15^N difference should decrease, reflecting the accumulation of newly produced, low-δ^15^N DON that is not balanced by consumption (Fig. 3 *B*, *Upper*). In contrast, in regions of net DON consumption, the labile DON-PN δ^15^N difference should increase above the steady-state value, reflecting isotopically fractionating DON degradation that is unbalanced by new, low-δ^15^N DON addition (Fig. 3 *B*, *Lower*). Thus, the δ^15^N difference between labile DON and PN should be lower in environments of net DON production and higher in environments of net DON consumption.

Comparison between the δ^15^N of suspended PN and labile DON in the Pacific basin reveals distinct regional distributions (Fig. 2 *D*-*F*). The δ^15^N of labile DON and PN converge in the regions of elevated nutrient supply (in the equatorial upwelling and eastern margin of the South Pacific), where DON concentrations are high, indicative of net DON production. Conversely, labile DON δ^15^N exceeds PN δ^15^N to a maximal degree in the subtropical gyres where DON concentrations are low, consistent with net DON consumption. As the PN data are sparse and since suspended PN δ^15^N is expected to covary with the δ^15^N of nitrate consumed in surface waters (*SI Appendix*, Fig. S6), we use the δ^15^N difference between labile DON and consumed nitrate (LDON-NO_3_^−^ Δδ^15^N) as a surrogate for the labile DON-PN δ^15^N difference, albeit with an offset. Consistent with this approach, the basin-scale distribution of LDON-NO_3_^−^ Δδ^15^N follows the spatial trends observed in labile DON-PN δ^15^N difference (*SI Appendix*, Fig. S7), providing a mechanistic explanation for the correspondence between DON concentration and LDON-NO_3_^−^ Δδ^15^N (*SI Appendix*, Fig. S5).

Thus, in the equatorial to temperate latitudes, the isotopic data provide observational evidence of net DON production in regions of high nutrient supply and net DON consumption in the nutrient-poor tropics and subtropics (13), with lateral transport of DON from the former to the latter (Fig. 3*B*, purple arrow). This is important, as spatial variations in DON concentration alone do not prove net DON transport among regions; they could be explained by a local environmental parameter (e.g., temperature, DON-producing phytoplankton biomass) that sets the DON concentration at which DON production and consumption are balanced. In this context, the DON isotope data provide critical confirmation that there are regions of net production, regions of net consumption, and net transport between them.

In contrast, as yet, the data do not support widespread DON transport from polar and subpolar waters to the subtropical gyres, as the polar and subpolar regions do not universally have lower labile DON δ^15^N compared to consumed nitrate δ^15^N (Fig. 2*D*); hence, a low LDON-NO_3_^−^ Δδ^15^N is not a consistent feature (*SI Appendix*, Fig. S7). This lack of high-latitude-to-subtropical transport may be due to the lack of an appropriate surface water advection pathway into the subtropical gyres. There may be net lateral supply from polar to subpolar and temperate regions, for example, from the Antarctic to Subantarctic Zone in the Southern Ocean (Fig. 2*D*, 40°–50°S), possibly due to westerly wind–driven Ekman transport (54, 55). However, the higher density of these higher latitude surface waters should limit their exchange with subtropical gyre surface waters. Of course, given our sparse spatial coverage, we may be missing regions hosting rapid high-to-low latitude net DON transport, such as may occur along the eastern sides of ocean basins.

**Fig. 4.**
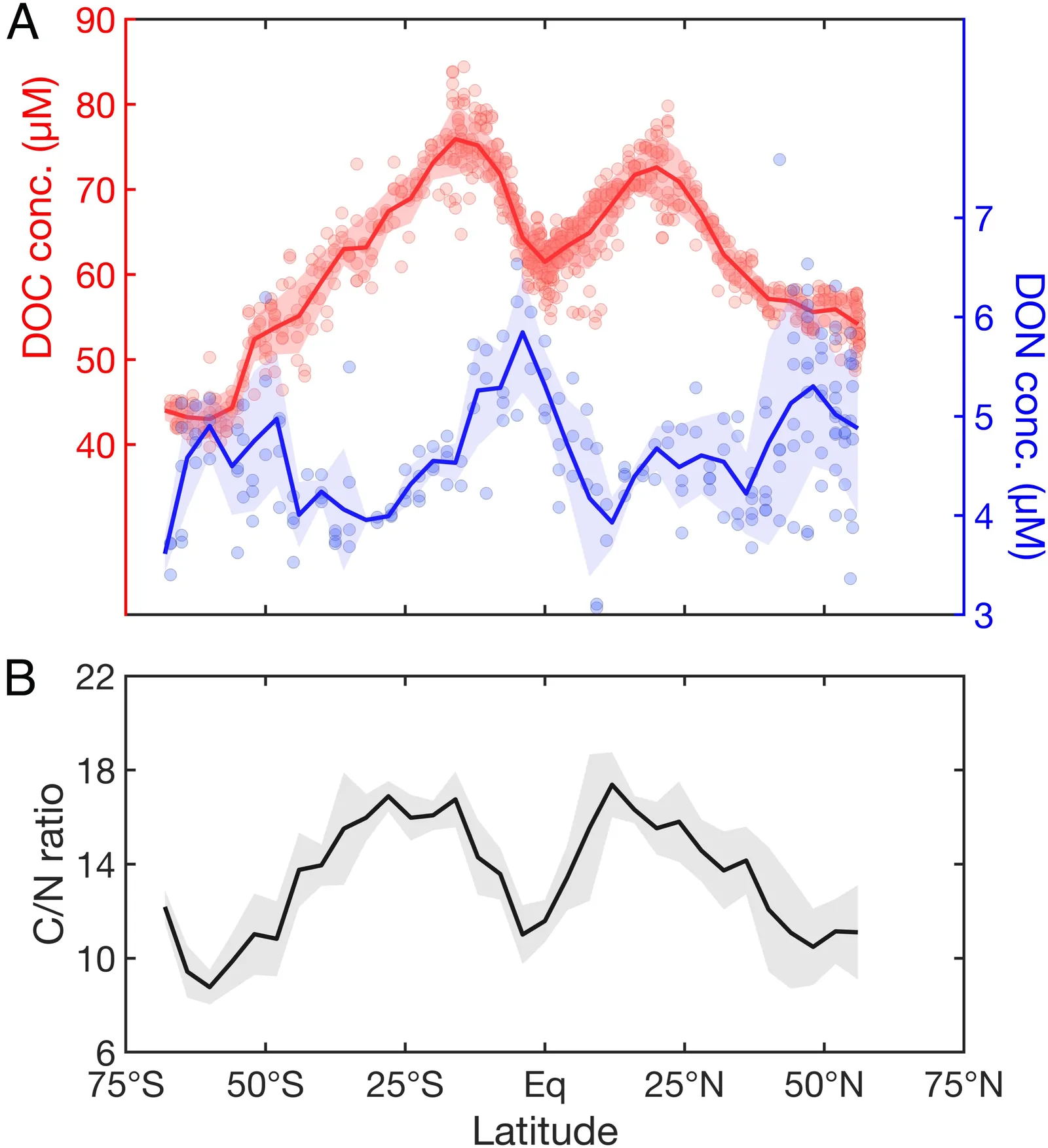
Meridional distribution of (*A*) DOC and DON concentrations and (*B*) DOC/DON ratio in the upper 100 m of the Pacific. In (*A*), red and blue circles represent DOC from the compilation (US GO-SHIP P16S and P16N transects only) (21) and DON from this study, respectively. Thick lines and shaded areas in (*A* and *B*) indicate the mean ± 1SD over a 4° grid (red: DOC, blue: DON, gray: DOC/DON ratio).

The role of DON as an organic nutrient is further supported by the contrast between the meridional distributions of surface DON and dissolved organic carbon (DOC) (Fig. 4*A*). While DON concentrations decrease from equatorial upwelling zones and subpolar regions toward the subtropical gyres, DOC concentrations exhibit the opposite trend. This results in a DOC-to-DON (C/N) ratio that is 9 to 11 in the equatorial upwelling zones and subpolar regions but up to ~17 in the subtropical gyres (Fig. 4*B*). This pattern suggests preferential microbial consumption of N-rich compounds in nutrient-limited surface waters, consistent with DON serving as a N source. In contrast, the high DOC concentrations in the subtropical gyres may originate from enhanced production and accumulation of DOC (56), possibly reflecting an excess in photosynthetic organic carbon production relative to the amount that can be used to build the key chemical constituents of phytoplankton biomass (57). In addition, both the accumulation of DOC and the drawdown of DON in the subtropical gyres may reflect N limitation of DOC-degrading microbes in these oligotrophic environments (58).

In general, our DON concentration and δ^15^N measurements suggest that low-latitude upwelling regions tend to host excess DON production, while subtropical gyres host DON consumption. However, the data also point to exceptions to this generalization, which we interpret in terms of nitrogen-specific processes. One case, described in the following section, involves the equatorial Atlantic. Here, we address a second case, which involves evidence of a difference between the northern and southern subtropical regions of the Pacific. Unlike other subtropical gyres, the subtropical North Pacific hosts labile DON that tends to be lower in δ^15^N than consumed nitrate, akin to the highly productive regions (*SI Appendix*, Fig. S7). This pattern might be explained by previous findings that N_2_ fixation constitutes a substantial fraction of new production in the subtropical North Pacific near Hawaii (35, 59), and that N_2_ fixers directly produce DON (50–52). An alternative possibility is that the DON has been imported from the western North Pacific gyre, where thermocline nitrate δ^15^N is lower, apparently due to a high fraction of newly fixed N relative to background nitrate (60). The observed smaller δ^15^N offset between PN and labile DON (and lower LDON-NO_3_^−^ Δδ^15^N values) in the western North Pacific gyre, together with elevated DON concentrations, provide further support for this interpretation (*SI Appendix*, Figs. S1 and S7). More broadly, the relatively high DON concentrations and low δ^15^N values in the subtropical North Pacific (23.5°–35°N) compared to the South Pacific (23.5°–35°S) (Fig. 1 *D* and *E*) are consistent with more N_2_ fixation leading to greater N availability in the former. As in the Pacific, the Atlantic is characterized by lower DON δ^15^N in the northern than the southern hemisphere (Fig. 1*I*), concordant with observations of high N_2_ fixation rates in the North (but not South) Atlantic (61). However, in the subtropical North Atlantic, DON δ^15^N does not appear to be consistently lower than the δ^15^N of the subsurface nitrate supply (*SI Appendix*, Fig. S8).

While N_2_ fixation is a source of low-δ^15^N N to the euphotic zone, we propose that its ability to lower the δ^15^N of DON in the subtropical North Pacific is partially, and perhaps mostly, due to its ability to cause net DON production (or reduce net DON consumption) in the region. As described above, when the production of DON outstrips the consumption of DON by isotopically fractionating DON remineralization, the δ^15^N of DON tends to be lower than the δ^15^N of the nitrate consumed in the euphotic zone (Fig. 3*A*). As for the view that the low δ^15^N of newly fixed N is the decisive driver of lower δ^15^N in the DON pool, it is undercut by the observation that there are other causes of a low δ^15^N in surface water PN, including partial nitrate assimilation (62) as well as the reassimilation of regenerated, low-δ^15^N ammonium (63, 64). Consequently, surface suspended PN generally has a low δ^15^N (37), and the breakdown and solubilization of this PN will contribute low-δ^15^N DON to the pool, regardless of whether N_2_ fixation is active.

### Net DON Degradation in the Equatorial Atlantic.

**Fig. 5.**
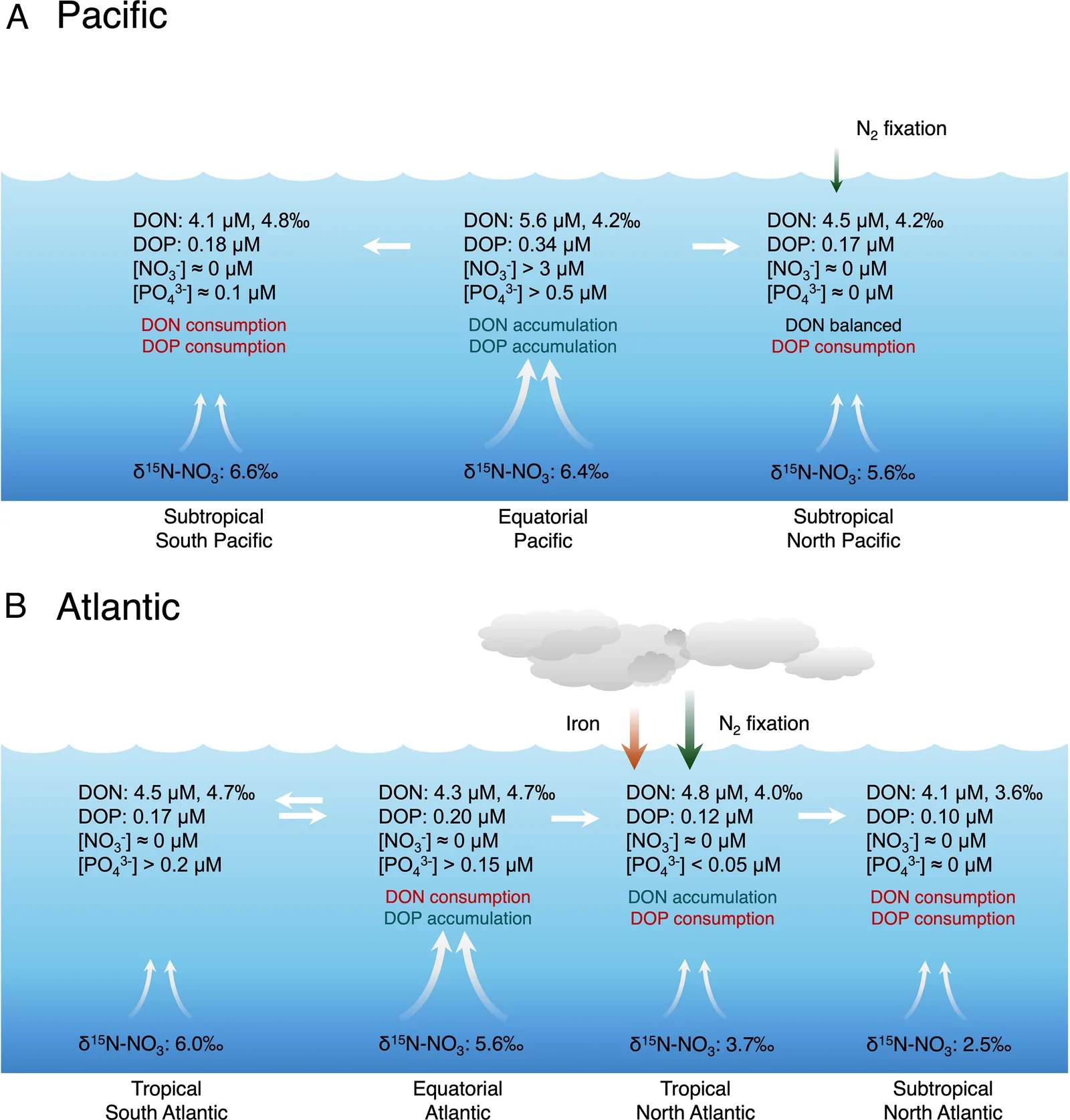
Variations in DON and DOP under different nutrient conditions across the (*A*) Pacific and (*B*) Atlantic basins. The figure illustrates the meridional evolution of surface DON concentration, δ^15^N of DON, DOP, nitrate and phosphate concentration, and subsurface nitrate δ^15^N. In (*A*), local N_2_ fixation and newly fixed N input from the western subtropical North Pacific are indicated with a green arrow, which is minor relative to the observed DON concentration decrease from the equatorial Pacific to the subtropical North Pacific. In (*B*), the influence of high aeolian iron input and N_2_ fixation in the North Atlantic is highlighted. The thickness of the white arrows indicates the intensity of nitrate supply from the subsurface.

In the Pacific, DON reaches a local concentration maximum in the equatorial upwelling zone, where net DON production is also evident from the lower δ^15^N DON values (Fig. 1 *D* and *E*). DOP also shows a local concentration maximum in this region (Fig. 1*G*) (65). The equatorial and tropical Pacific receive upwelled waters containing phosphate and nitrate, which largely remain unused in surface waters, indicating that N and P are not limiting for biological production in this region. Under these N- and P-replete conditions, DON and DOP are likely to accumulate at the surface. Downstream of the upwelled waters, DON and DOP are consumed in the subtropical gyres, where vertical nutrient supply is highly limited (Fig. 5*A*), leading to a generally coherent latitudinal variation in DON and DOP. As described above, in the subtropical North Pacific, N_2_ fixation may reduce the pressure for DON consumption (and associated DON δ^15^N elevation).

In contrast, in the equatorial to tropical North Atlantic, DON and DOP are decoupled. At the equator in the Atlantic, surface DON is relatively low and DON δ^15^N is slightly elevated, especially to the northwest downstream of the equatorial upwelling (Fig. 1 *H* and *I*). The elevated DON δ^15^N is not due to Amazon River inputs, which appear to be associated with a relatively low DON δ^15^N (~3.5‰) (11). Instead, these data point to net DON consumption. In contrast, DOP concentration has no apparent equatorial minimum (Fig. 1*K*). Moving toward the tropical North Atlantic, the DON concentration increases while the DOP concentration decreases. This transition coincides with the northward increase in iron availability (*SI Appendix*, Figs. S2 and S9) due to relatively low dust-borne iron supply in the equatorial Atlantic and a much greater supply over the tropical and subtropical North Atlantic (61, 66). DON δ^15^N undergoes a ~1‰ northward decline from the equatorial to the subtropical North Atlantic, in step with a ~3‰ northward decline in the δ^15^N of shallow subsurface nitrate. This low-δ^15^N signal may partially reflect contributions from atmospheric N inputs (24–26). At the basin scale, however, N_2_ fixation is likely the dominant source of new N to the North Atlantic, and it occurs primarily in the tropical North Atlantic (61), so it probably explains most of the northward decrease in the δ^15^N of tropical North Atlantic thermocline nitrate (67). Thus, the combined concentration and isotopic data point to N_2_ fixation in the tropical North Atlantic, further downstream of the equatorial Atlantic, stimulated by the simultaneous availability of P (as phosphate and DOP) and iron. The rise in DON concentration across the tropical North Atlantic (Fig. 1*H*) suggests either that the N_2_ fixers produce DON or that the N_2_ fixation-fueled increase in N availability and productivity causes other plankton to increase their DON production or decrease their DON consumption.

The South Atlantic and the Atlantic equatorial upwelling zone are relatively P-rich, with iron supply being low yet sufficient for phytoplankton to completely draw down upwelled nitrate (*SI Appendix*, Figs. S2 and S9). As a result, nitrate is completely depleted in the surface waters, giving rise to a basin-wide N-limited environment. This stands in contrast to the Pacific, where a greater nutrient upwelling rate and persistent iron limitation prevents complete consumption of either phosphate or nitrate, allowing DON accumulation in and near the equatorial Pacific (Fig. 5*A*). Motivated by this contrast between the equatorial Atlantic and Pacific, we propose that the meridional changes in DON concentration and δ^15^N in the equatorial Atlantic identify net DON consumption as a pathway by which excess N is supplied to an ecosystem limited by N but replete in P, providing an alternative to N_2_ fixation under the low-iron conditions (Fig. 5*B*). Meanwhile, the P richness appears to suppress DOP consumption, consistent with the lack of a decline in DOP concentration in the equatorial Atlantic (Fig. 1*K*) (17). Photochemical reactions in the surface ocean may also drive DON degradation (68). However, these processes would likely have a broad geographic impact and cannot account for the localized DON concentration minimum and δ^15^N maximum observed in the equatorial Atlantic. Moreover, photochemical degradation would not cause the selective decomposition of DON relative to DOP, making the observed pattern of a DON minimum and DOP maximum inconsistent with this mechanism. Taken together, these observations support microbial (i.e., bacterial) degradation as the primary mechanism for DON loss. These findings raise the possibility that microbial degradation pathways are adapted to local nutrient regimes, selectively targeting DON or DOP depending on regional N, P, and iron availability. Under a putative ongoing shift away from N limitation and toward P limitation (69), microbial consumption of DON is likely to be reduced, which may cause DON concentrations to rise in surface waters.

### Subtropical Biological Production Fueled by DON.

Overall, our findings indicate that ~25% of the DON in the upper ocean undergoes active local production and consumption on timescales comparable to the horizontal circulation of surface waters, on the order of months, which is longer than phytoplankton turnover timescales of a week (70). The temporal decoupling allows this labile DON, delivered via lateral transport, to act as a buffer for primary production, sustaining productivity in the oligotrophic gyres despite their lack of DIN during most of the year.

In the Sargasso Sea, surface dissolved inorganic carbon (DIC) drawdown begins in early spring and continues through late summer to early fall, despite near-complete DIN depletion after May (20, 71). The continued DIC drawdown has been attributed to net community production (72), which is unlikely to be sustained solely by nitrate supply (73) or N_2_ fixation (67). The δ^15^N of labile DON in the Sargasso Sea is significantly elevated relative to that of subsurface nitrate (i.e., has a high LDON-NO_3_^−^ Δδ^15^N; *SI Appendix*, Fig. S8). This δ^15^N elevation in labile DON indicates that the Sargasso Sea acts as a region of net DON consumption that receives and utilizes laterally transported DON. Consistent with this, DON concentrations exhibit a sharp decrease of 0.9 ± 0.4 µM from the boundary of the subtropical North Atlantic gyre into the Sargasso Sea (Fig. 1*H*). Combining this decrease with the Ekman downwelling rate for the Sargasso Sea (0.11 ± 0.05 m d^−1^; 20°–35°N, 40°–70°W; NOAA ERDDAP data from 2021 to 2025), we estimate an annual lateral advective DON supply of 0.04 ± 0.02 mol N m^−2^ y^−1^. This lateral advective DON flux alone can account for only a modest fraction (~10%) of the total N demand for net community production in the Sargasso Sea (~0.45 ± 0.15 mol N m^−2^ y^−1^, converted from 3.0 ± 1.0 mol C m^−2^ y^−1^, assuming the canonical Redfield C:N ratio of 6.6) (74). However, this calculation does not account for horizontal diffusive fluxes (or eddies), which may significantly increase the net transport of DON into the subtropical gyres (75). With more work to quantify the isotope effect of DON remineralization (12, 13; *SI Appendix*, Fig. S10), coupled DON concentration and isotope measurements could improve quantification of regional DON production and consumption.

## Materials and Methods

### Seawater Sampling.

Seawater samples were collected in 20, 30, or 60 mL HDPE bottles. For the US GO-SHIP projects (P02 2013, P06 2017, A16N 2013, A13.5 2020, A20/A22 2021), the *R/V Brown* cruise in the equatorial Pacific, and Station K1, HDPE bottles were prewashed with 10% acid and rinsed at least three times with sample water before use. Except for P06 samples, which were filtered with syringe filters, these samples were collected unfiltered, with no particles removed. In contrast, samples from the GEOTRACES projects (GP15, GP17-OCE) were filtered through 0.45 µm pore-size filters to remove particles. For these GEOTRACES samples, HDPE bottles were not acid-washed prior to use; instead, each bottle was rinsed three times with sample water before being filled to the shoulder. All samples were stored frozen at −20 °C.

### δ^15^N Measurements of Nitrate and DON.

The δ^15^N of seawater nitrate was measured using the denitrifier method (76), which encompasses both nitrate and nitrite, although the contribution of nitrite is minor. In this method, nitrate is quantitatively reduced to nitrous oxide (N_2_O) using a strain of denitrifying bacteria, *Pseudomonas* chlororaphis ssp. aureofaciens (ATCC 13985, Manassas, VA), which lacks nitrous oxide reductase activity. The nitrogen isotopic composition of the resulting N_2_O was analyzed using a custom-built N_2_O extraction and purification system (76–79) online to a Thermo MAT253 isotope ratio mass spectrometer. Calibration for δ^15^N of nitrate was performed using two international reference standards: USGS34 (potassium nitrate, δ^15^N = −1.8‰) and IAEA-N3 (potassium nitrate, δ^15^N = 4.7‰).

The concentration of DON was calculated by subtracting the nitrate concentration from the total dissolved nitrogen (TDN) concentration in the original samples: [DON] = [TDN] − [NO_3_^−^]. Nitrate concentrations (µmol kg^−1^) were converted to µM using seawater density at 20 °C. The oxidation of organic nitrogen to nitrate for isotopic analysis was achieved via persulfate oxidation, following the protocol of ref. 9 and the method of ref. 13. In a precombusted 4 mL glass vial, 2 mL of seawater was mixed with 2 mL of a persulfate oxidizing reagent (POR) consisting of 1 g K_2_S_2_O_8_ and 1.5 g NaOH dissolved in 100 mL of low-nitrogen purified water. The mixture was heated to 140 °C for 1 h, after which the pH was adjusted to near neutral. The nitrate produced through oxidation was then analyzed for δ^15^N using the denitrifier method, employing a different strain of *Pseudomonas* chlororaphis (ATCC 43928, Manassas, VA).

The isotopic composition of DON was calculated using the following equation: δ^15^N DON = [(δ^15^N TDN × [TDN]) − (δ^15^N NO_3_ × [NO_3_^−^])]/[DON]. Minor nitrogen species, including ammonium, were excluded from the calculation because they are present at submicromolar concentrations in most open-ocean surface waters (80). Suspended PN was also excluded as it is typically present at submicromolar concentrations in surface waters (37, 81). The 1SD for TDN concentration and δ^15^N measurements were, on average, 0.2 µM and 0.2‰. For DON concentration and δ^15^N obtained from the TDN differencing method across the entire sample set, the corresponding average SDs were 0.3 µM and 0.4‰, respectively.

## Supplementary Material

Appendix 01 (PDF)

## Data Availability

The DON concentration and δ^15^N data and the nitrate δ^15^N data used in this study have been deposited in Zenodo (10.5281/zenodo.15116364) (82); the full nitrate isotope data sets will also be submitted to CCHDO and BCO-DMO. Hydrographic and nitrate concentration data used to calculate DON were obtained from the US GO-SHIP cruises P02 (2013), P06 (2017), A16N (2013), A20 (2021), A22 (2021), and A13.5 (2020) via the CLIVAR and Carbon Hydrographic Data Office (CCHDO; https://cchdo.ucsd.edu), and from the US GEOTRACES GP15 (2018) and GP17-OCE (2022–2023) cruises via the Biological and Chemical Oceanography Data Management Office (BCO-DMO) (83–87).
