# Supplementary material for: Isotopic evidence of dissolved organic nitrogen production and consumption in ocean surface waters
Source: Proc Natl Acad Sci U S A. 2026 Oct 28;123(44):e2530315123. doi: 10.1073/pnas.2530315123 (PMC13732207; doi:10.1073/pnas.2530315123)
Supplement: Supplementary file 1 [file pnas.2530315123.sapp.pdf]

Supporting Information for

Isotopic evidence of dissolved organic nitrogen production and consumption in ocean surface waters

Yeongjun Ryu[1,2*], Dario Marconi[1], Zhou Liang[3], Angela N. Knapp[4], François Fripiat[5], Sarah E. Fawcett[6,7], Patrick A. Rafter[8], Sergey Oleynik[1], Alfredo Martínez-García[2], Gerald H. Haug[2,9], Daniel M. Sigman[1]

[1]Department of Geosciences, Princeton University, Princeton, New Jersey, USA
[2]Climate Geochemistry Department, Max Planck Institute for Chemistry, Mainz, Germany
[3]Division of Biosphere Sciences and Engineering, Carnegie Institution for Science, Pasadena, California, USA
[4]Oceanography Department, Texas A&M University, College Station, Texas, USA
[5]Department of Geosciences, Environment and Society, Université Libre de Bruxelles, Brussels, Belgium
[6]Department of Oceanography, University of Cape Town, Rondebosch, South Africa
[7]Marine and Antarctic Research Centre for Innovation and Sustainability, University of Cape Town, Rondebosch, South Africa
[8]College of Marine Science, University of South Florida, St Petersburg, Florida, USA
[9]Department of Earth and Planetary Sciences, ETH Zürich, Zürich, Switzerland

* Corresponding author: Yeongjun Ryu
Email: yeongjun@princeton.edu

This PDF file includes:

Supporting text
Figures S1 to S11
SI References

Supporting Information Text

Vertical distribution of DON at the BATS station

Depth profiles of DON concentration and $\delta^{15}N$ at the BATS station (32°10'N, 64°30'W) were determined by subtracting nitrate from total nitrogen (TN) (Fig. S4). Water samples were collected in acid-washed HDPE bottles during the August 2022 BATS cruise (33H420220803) without filtration and stored at –20°C until analysis. Nitrate is completely depleted in the upper 100 m of waters, while DON concentrations average 4.2 ± 0.2 μM. DON concentrations decrease sharply in the upper thermocline and then stabilize below 1000 m at 2.9 ± 0.4 μM.
In contrast, the $\delta^{15}N$ of DON increases with depth. In the upper 100 m, DON $\delta^{15}N$ averages 3.1 ± 0.3‰. It rises sharply within the upper thermocline before stabilizing below 1000 m at 3.9 ± 0.4‰. The mean deep-water DON concentration and $\delta^{15}N$ values (≥1000 m) are used in the main text as representative of the non-labile DON pool in the two-component model.

Although the division into just two DON pools, labile and non-labile, is arbitrary, the chemically distinctive structure of deep oceanic DON (1) and its refractory nature provide a rationale for treating the deep Atlantic DON as broadly representative of global ocean non-labile DON. Deep Atlantic waters contain substantially lower nitrate concentrations (~20 μM) than the Pacific (~40 μM), which allows more reliable DON $\delta^{15}N$ measurements, given that DON is obtained by subtracting nitrate from TDN and that the associated analytical uncertainties increase with nitrate concentration (see Methods). For these reasons, the Atlantic deep DON values were adopted as the primary estimate for the non-labile endmember. Previous studies have reported modest inter-basin differences. Total DON concentrations are slightly lower in the deep North Pacific (2.4 μM) (2) compared to the Atlantic (2.9 ± 0.4 μM), whereas solid-phase extracted DON measurements suggest modestly higher $\delta^{15}N$ (4.2‰) in the deep North Pacific (1), and similar gradients may also exist in total DON $\delta^{15}N$. However, sensitivity tests assuming the deep North Pacific DON as the non-labile endmember did not qualitatively alter the overall basin-scale patterns of LDON-NO_3^- $\Delta\delta^{15}N$ (Fig. S11).

Two-component model for surface DON

The strong correlation (r^2=0.63) between the $\delta^{15}N$ of surface DON and the $\delta^{15}N$ of subsurface nitrate in regions where surface nitrate is depleted (<1 μM; Fig. 2A) suggest that isotopic composition of surface DON is influenced by nitrate supply from the subsurface. Given that the subsurface nitrate pool is much larger than all other forms of N, the $\delta^{15}N$ of subsurface nitrate primarily controls the $\delta^{15}N$ of surface organic N rather than the reverse (3,4). Thus, the observed $\delta^{15}N$ correlation most likely reflects a response of surface DON to nitrate supplied to and consumed in the surface ocean.

The relatively narrow range of variation in surface DON $\delta^{15}N$ than nitrate suggests that, in general, may consist of two components: a highly labile DON fraction produced from the subsurface nitrate supply, and a uniform, non-labile DON fraction distributed throughout the global ocean.

In this two-component model, assuming a uniform concentration and $\delta^{15}N$ value for non-labile DON across the ocean, we can write mass balance equations for the observed total DON pool with labile (DON_{labile}) and non-labile ($DON_{non\text{-}labile}$) components.

$$[DON] = [DON]_{labile} + [DON]_{non\text{-}labile}$$
$$\delta^{15}N\text{-}DON*[DON] = \delta^{15}N\text{-}DON_{labile}*[DON]_{labile} + \delta^{15}N\text{-}DON_{non\text{-}labile}*[DON]_{non\text{-}labile}$$

Here, we use the DON concentration and $\delta^{15}N$ of deep water (below 1000 m) at the BATS station to represent non-labile DON (2.9 ± 0.4 μM and 3.9 ± 0.4‰, respectively; Fig. S4). We can thus solve the equation for the $\delta^{15}N$ of labile DON, as follow:

$$\delta^{15}N\text{-}DON_{labile} = (\delta^{15}N\text{-}DON*[DON] - \delta^{15}N\text{-}DON_{non\text{-}labile}*[DON]_{non\text{-}labile})/(1 - [DON]_{non\text{-}labile})$$

We then compare $\delta^{15}N\text{-}DON_{labile}$ with the subsurface nitrate $\delta^{15}N$ (Fig. 2B), and with consumed nitrate $\delta^{15}N$ (Fig. 2C).

Calculation of the $\delta^{15}N$ of consumed nitrate

To evaluate the isotopic relationship between labile DON and the inorganic nitrogen source utilized by primary producers, we estimated the $\delta^{15}N$ of the nitrate consumed by phytoplankton in the surface ocean. Assuming that the deficit in nitrate concentration between the subsurface and the surface is entirely driven by biological drawdown, the concentration of consumed nitrate ($[NO_3^-]_{consumed}$) within the upper water column can be defined as:

$$[NO_3^-]_{consumed} = [NO_3^-]_{subsurface} - [NO_3^-]_{surface}$$

where the subscripts "surface" and "subsurface" represent the mean values for the upper 100 m and the 200–300 m depth intervals, respectively. Based on a conservative isotopic mass balance, the N isotope budget for the upper water column is expressed as follows:

$$\delta^{15}N_{consumed}*[NO_3^-]_{consumed} = \delta^{15}N_{subsurface}*[NO_3^-]_{subsurface} - \delta^{15}N_{surface}*[NO_3^-]_{surface}$$

Rearranging this equation yields the $\delta^{15}N$ of the consumed nitrate ($\delta^{15}N_{consumed}$):

$$\delta^{15}N_{consumed} = (\delta^{15}N_{subsurface}*[NO_3^-]_{subsurface} - \delta^{15}N_{surface}*[NO_3^-]_{surface})/[NO_3^-]_{consumed}$$

In regions where nitrate consumption in the surface layer is complete, the equation simplifies to:

$$\delta^{15}N_{consumed} = \delta^{15}N_{subsurface}$$

Under this complete drawdown case, the $\delta^{15}N$ of the nitrate assimilated by phytoplankton is equivalent to the $\delta^{15}N$ of the source nitrate supplied from the subsurface.

Estimating the isotope effect of DON degradation

To estimate the isotope effect for DON degradation, vertical profiles from the upper 250 m were used. This approach minimizes the influence of different water masses (i.e., thermocline and deep waters) and better isolates the consumption signal as DON produced at the surface is remineralized in the subsurface, allowing independent estimates of isotope effects without spatial biases. For each profile, the slope of log(DON concentration) versus DON $\delta^{15}N$ was calculated using linear regression, and slopes with t-statistics >2 were considered statistically significant.

Regional differences were observed: some areas exhibited negative slopes, while others showed positive slopes. Negative slopes were mostly found in productive regions, where DON $\delta^{15}N$ generally increases (and DON concentration decreases) monotonically with depth. In contrast, positive slopes were mostly observed in subtropical regions, where DON $\delta^{15}N$ typically shows a subsurface minimum and increases again below it. These subsurface DON $\delta^{15}N$ minima coincide with subsurface nitrate $\delta^{15}N$ minima, possibly indicating other processes in the lower euphotic zone. Therefore, these positive slopes were excluded from further analysis.

Among the statistically significant slopes (n=38), a weighted mean, accounting for standard errors, was calculated as 2.2 ± 0.4‰ (95% confidence interval). This value is comparable to isotope effects reported for the degradation of other organic nitrogen compounds (5,6) but smaller than previous estimates for DON degradation (~5‰) (7,8).

Figures and Tables

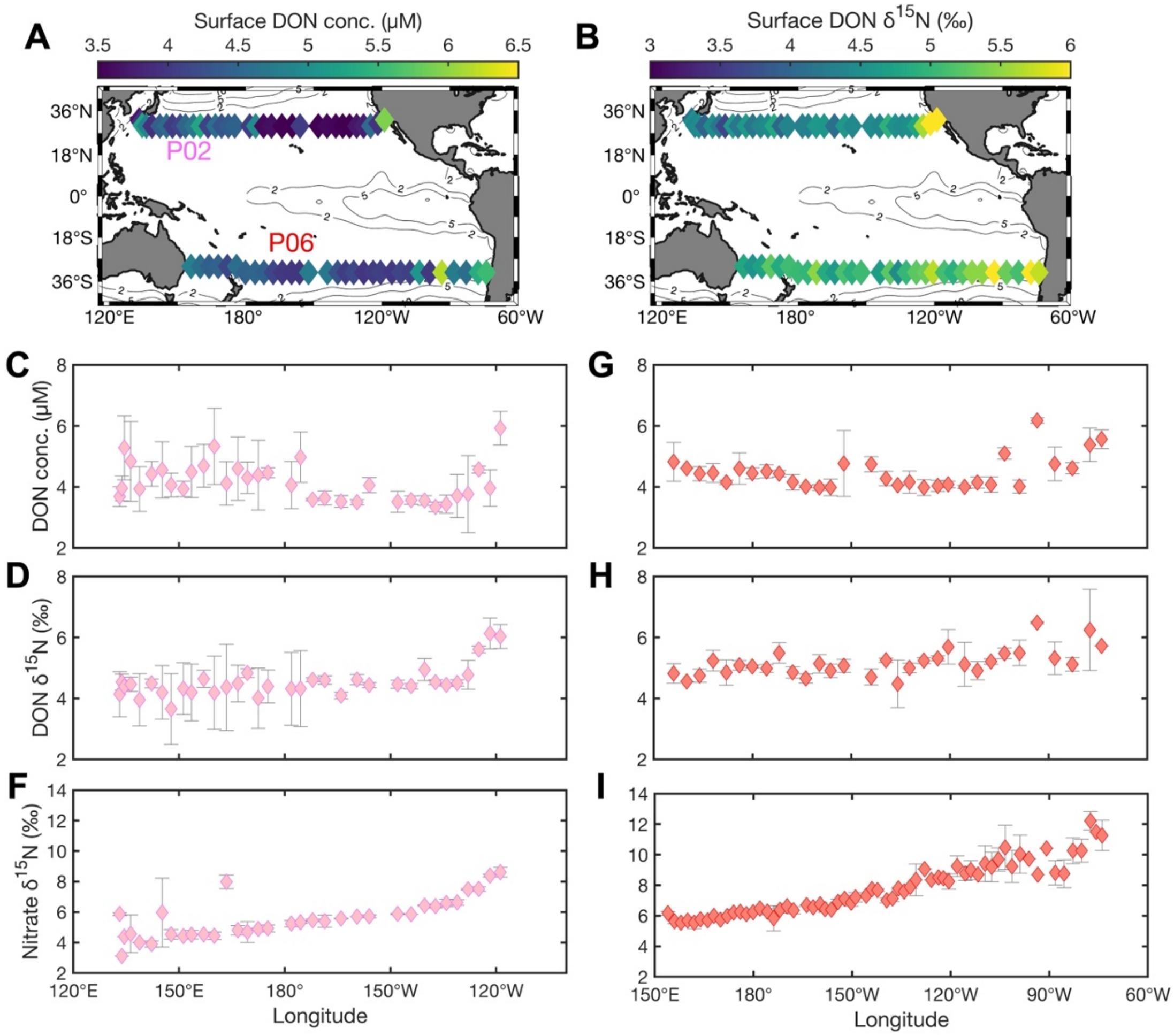

Fig. S1. Zonal distribution of DON concentration and δ^{15}N in the subtropical North and South Pacific. Maps with symbols show surface mean (upper 100 m) (**A**) DON concentration and (**B**) DON δ^{15}N measurements over P02 (~30°N) and P06 (~30°S) transects. Panels (**C–I**) show zonal distributions of (C,G) DON concentration, (D,H) DON δ^{15}N averaged over the upper 100 m, and (F,**I**) subsurface nitrate δ^{15}N (200–300 m). Pink and red symbols indicate samples from the subtropical North and South Pacific, respectively. Symbols represent depth-averaged values, and error bars denote ± 1SD. Panels (C–F) correspond to the subtropical North Pacific, and panels (G–I) to the subtropical South Pacific.

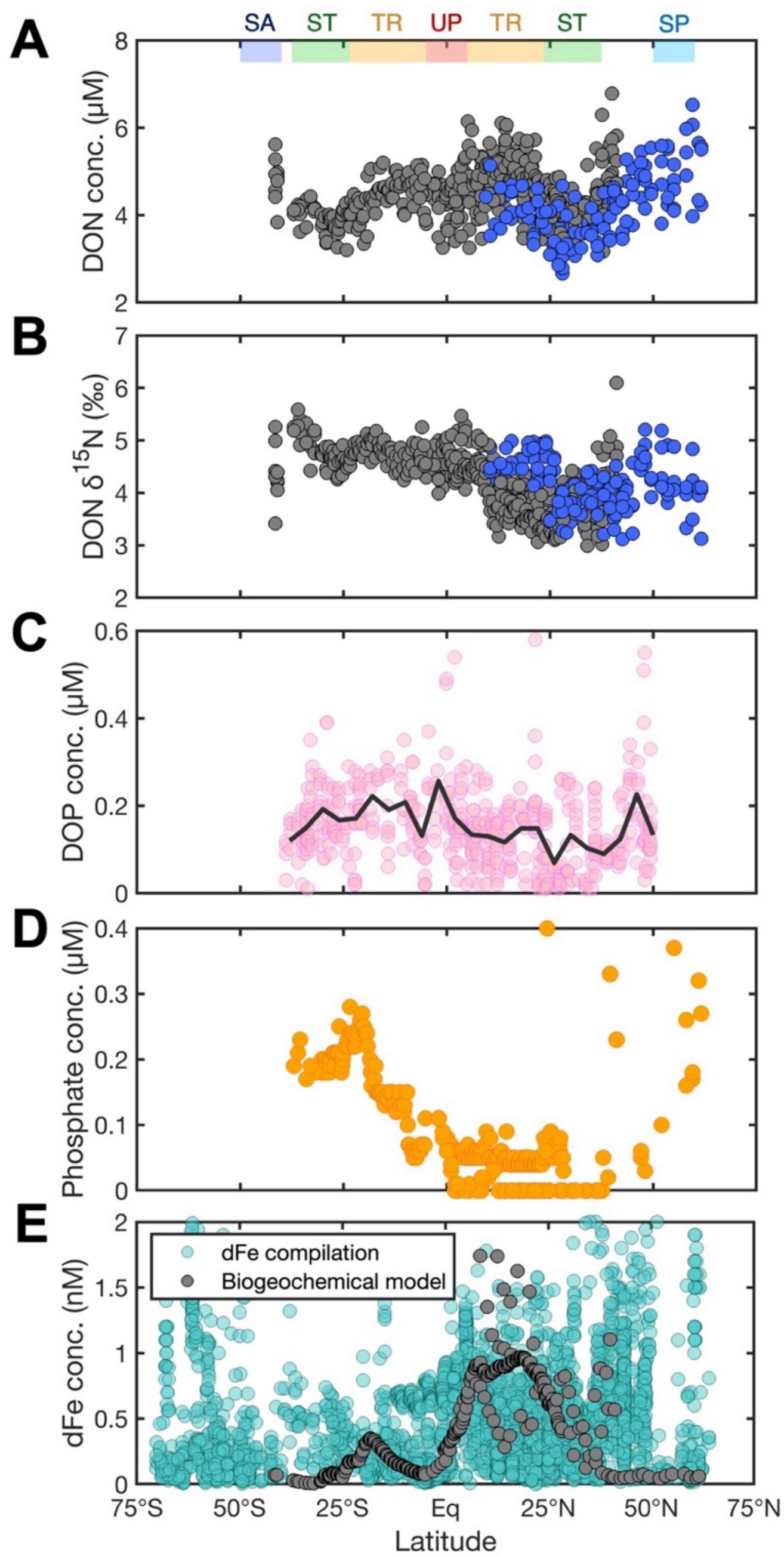

Fig. S2. Latitudinal variation in surface dissolved organic nutrients and nutrient conditions across the Atlantic basin. The upper three panels ((**A**) DON concentration, (**B**) DON $\delta^{15}N$, and (**C**) DOP concentration) correspond to those in Fig. 1. In (A,B), DON data from the eastern North Atlantic transect of A16N, north of 9°N, are denoted as blue circles; these are excluded from Fig. 1. (**D**) Phosphate concentration in surface water. (**E**) Dissolved iron (dFe) concentration in upper 100 m, derived from a compiled measurement dataset (cyan circles) (9) and surface water dFe from biogeochemical model outputs over the sampling locations (Copernicus Marine Service Information (ref. 10)).

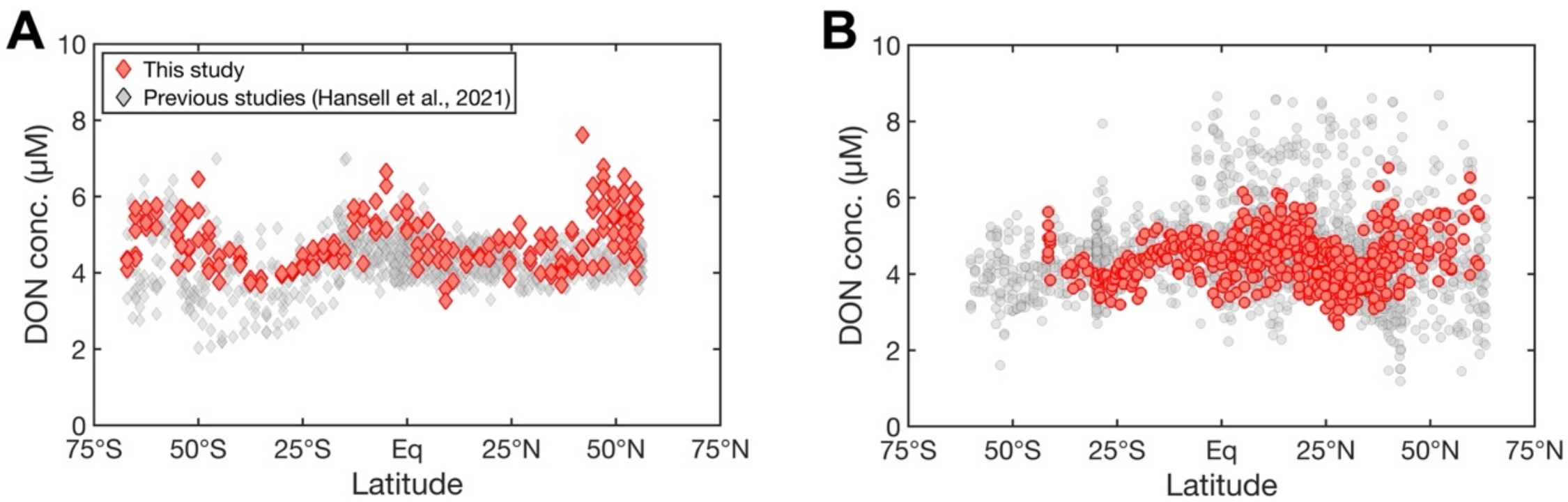

Fig. S3. Comparison of new DON data with previous measurements in the (A) Pacific (GP15 and GP17-OCE) and (B) Atlantic. Red symbols represent DON data from this study, while gray symbols indicate data compiled from previous studies (11). To enable a direct comparison, we subsampled cruise transect data from regions geographically close to our sampling sites (US GO-SHIP P16N and P16S for the Pacific; A13.5, A16S, A16N, A20, and A22 for the Atlantic).

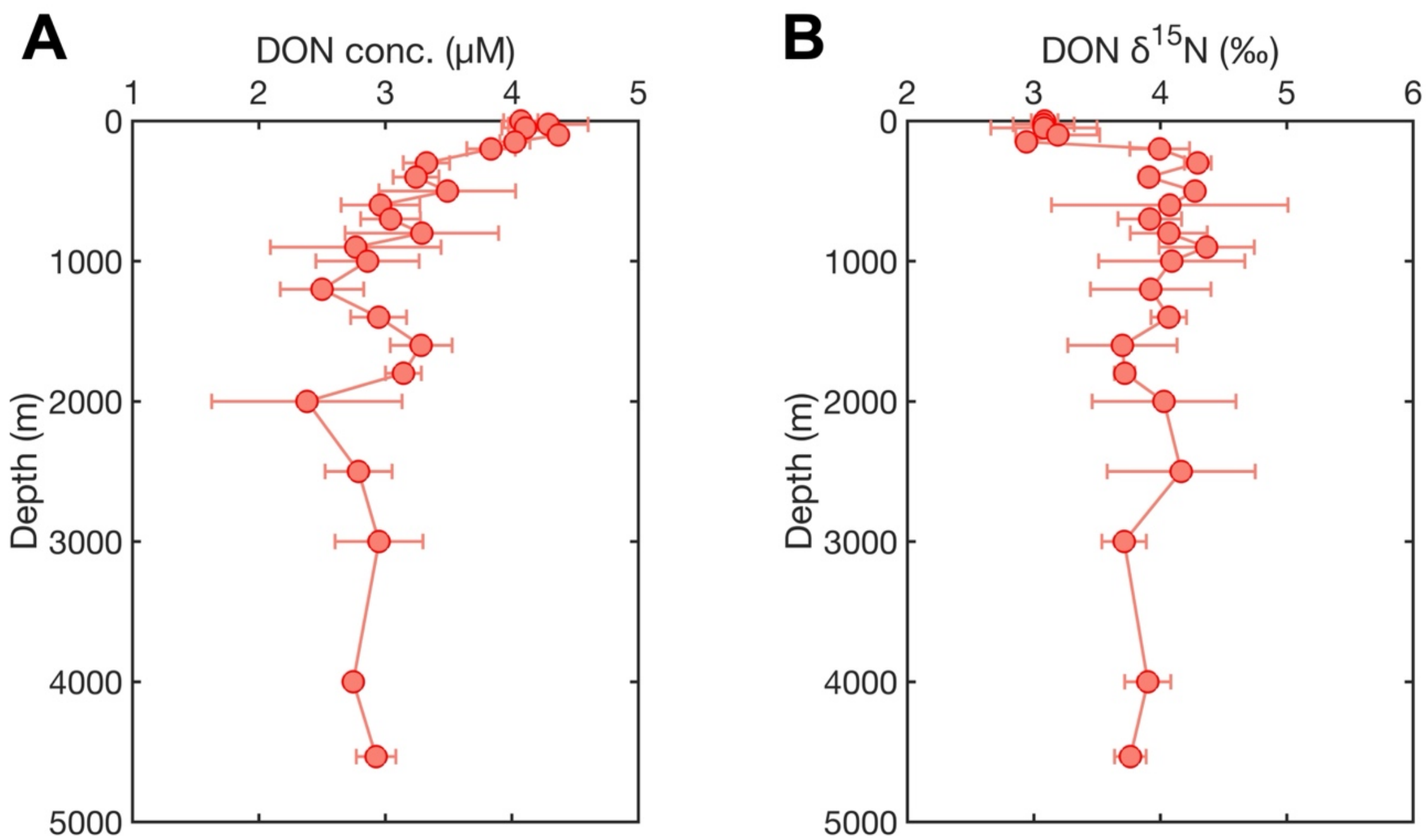

Fig. S4. Vertical profiles of (A) DON concentration and (B) $\delta^{15}N$ at the BATS station. Error bars represent ± 1SD of replicate measurements.

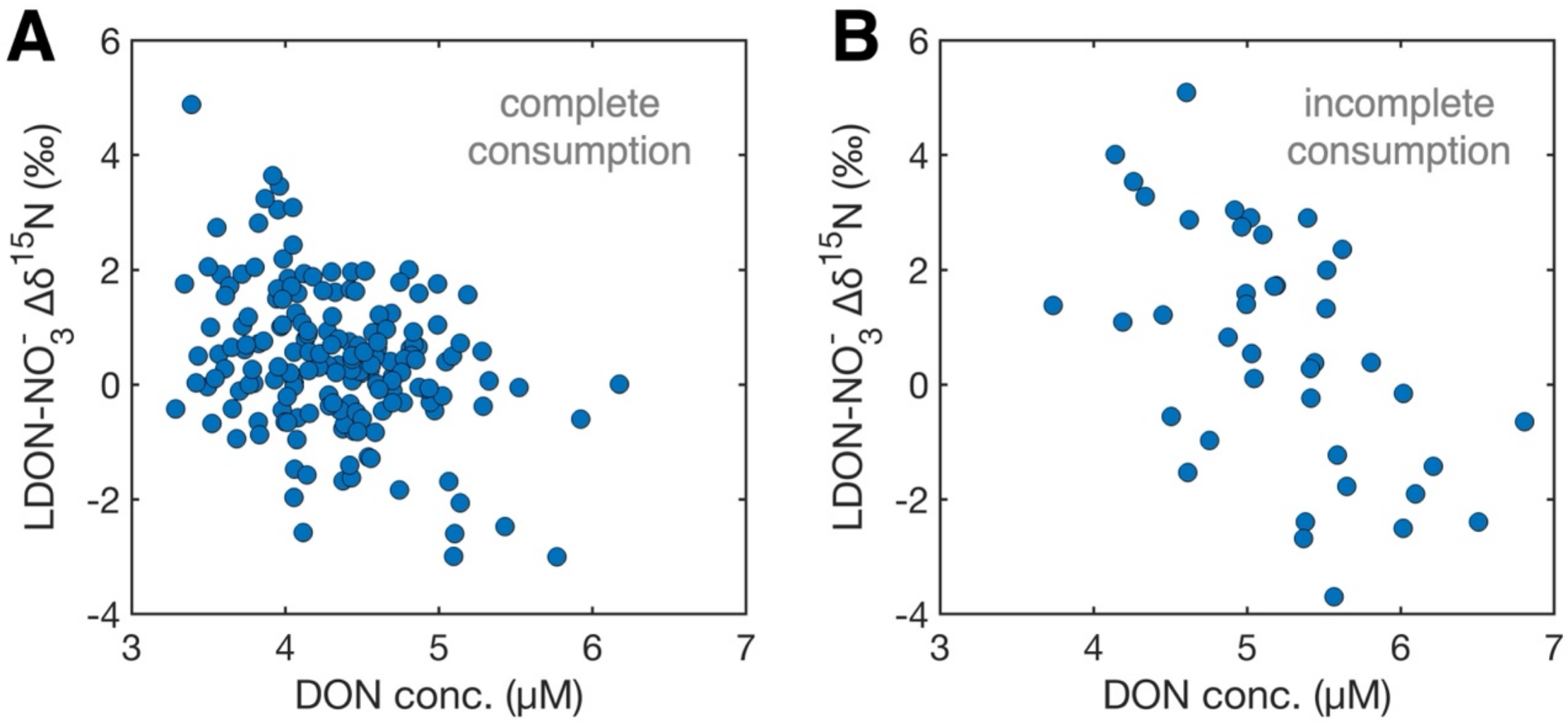

Fig. S5. Relationship between surface DON concentration and LDON-NO_3^- $\Delta\delta^{15}N$. (**A**) Samples from locations where nitrate is completely consumed in the surface ocean ($[NO_3^-]<1$ μM), and (**B**) samples from locations where nitrate remains in the surface ocean ($[NO_3^-]>1$ μM). LDON-NO_3^- $\Delta\delta^{15}N$ is defined as the difference between labile DON $\delta^{15}N$ and consumed nitrate $\delta^{15}N$, where consumed nitrate $\delta^{15}N$ was calculated from surface to subsurface nitrate difference ($\delta^{15}N_{consumed} = (\delta^{15}N_{subsurface}*[NO_3^-]_{subsurface} - \delta^{15}N_{surface}*[NO_3^-]_{surface})/[NO_3^-]_{consumed}$).

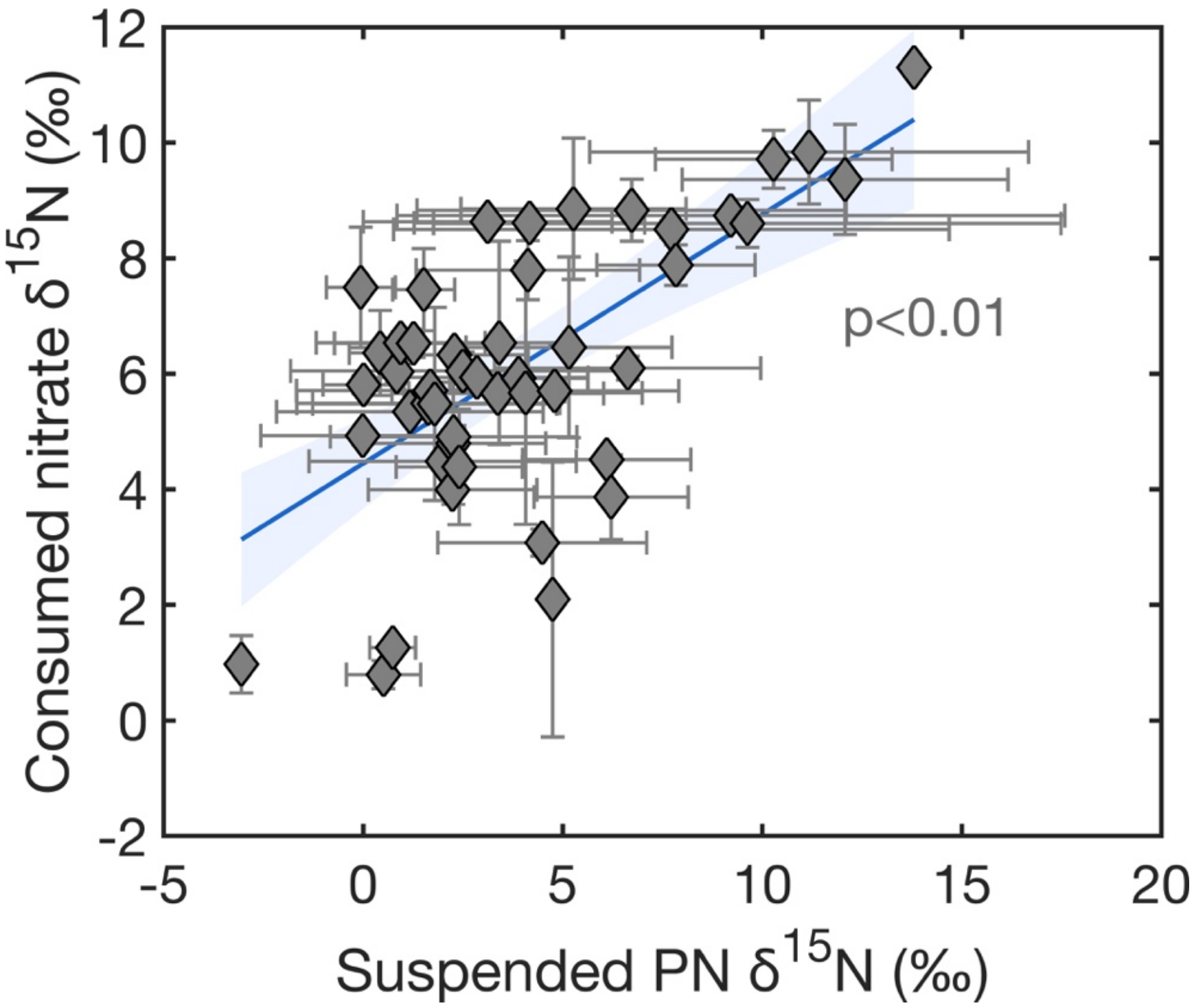

Fig. S6. Relationship between consumed nitrate $\delta^{15}N$ and suspended PN $\delta^{15}N$ across the Pacific. The blue solid line indicates the linear regression, and the light blue shading denotes the 95% confidence interval ($p<0.01$). The consumed nitrate and suspended PN data are the same as those shown in Figs. 2D-F. Data from the meridional transects (GP15 and GP17-OCE; Fig. 2D) and zonal transects (P02 and P06; Figs. 2E,F) were averaged within 5° and 10° latitude/longitude grid bins, respectively. Error bars represent ± 1SD.

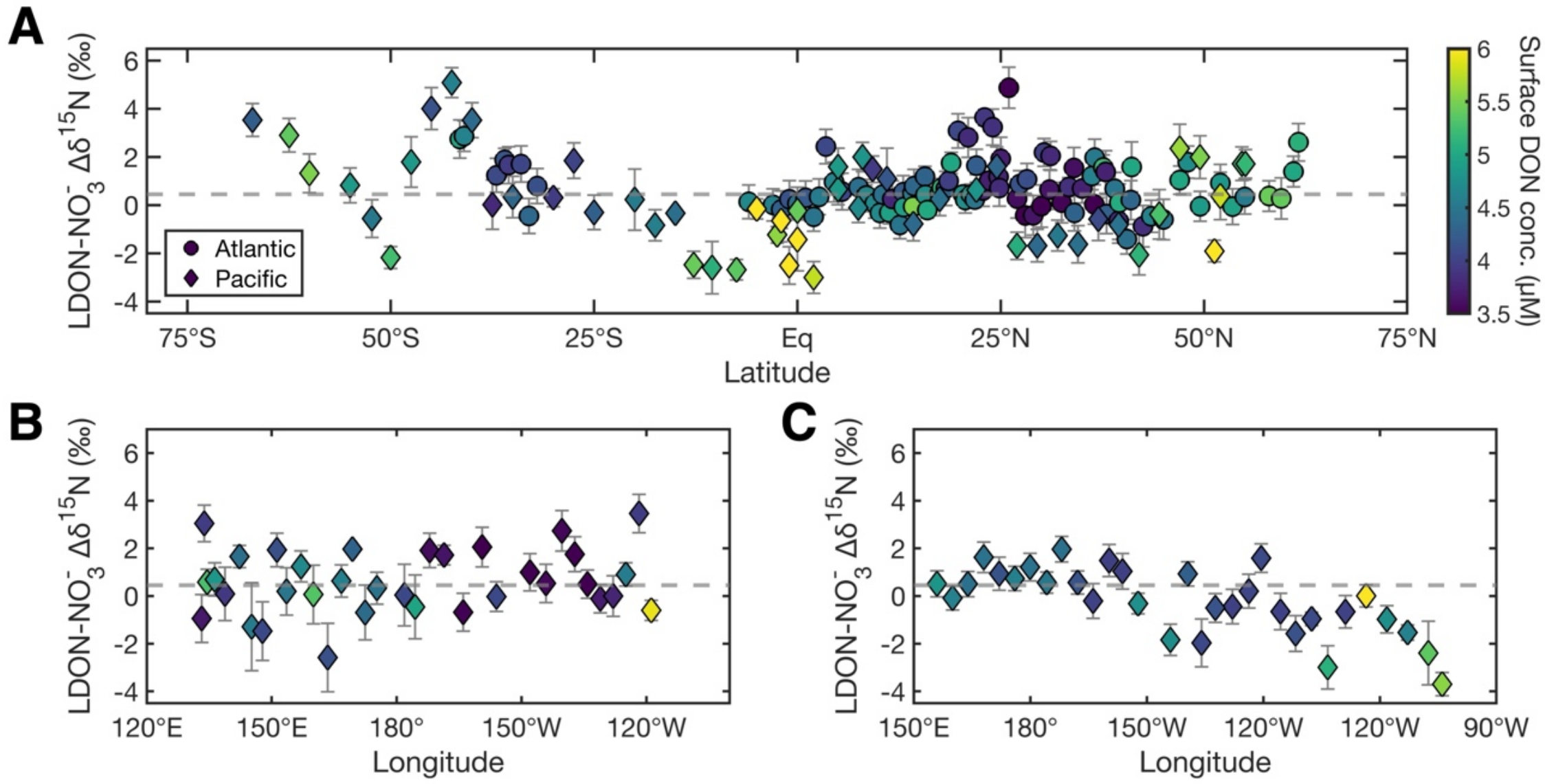

Fig. S7. Meridional and zonal distributions of LDON-NO_3^- $\Delta\delta^{15}N$. (**A**) Meridional transects across the Atlantic and Pacific Oceans (Atlantic: A16N, A20, A22, and A13.5; Pacific: GP15 and GP17-OCE), where circles and diamonds represent data from the Atlantic and Pacific, respectively. (**B**) Zonal transect across the subtropical North Pacific (P02). (**C**) Zonal transect across the subtropical South Pacific (P06). Symbols with error bars indicate mean values ± 1SD and are color-coded by surface DON concentration. In all panels, the horizontal gray lines denote the mean across the datasets (LDON-NO_3^- $\Delta\delta^{15}N$ = 0.5‰).

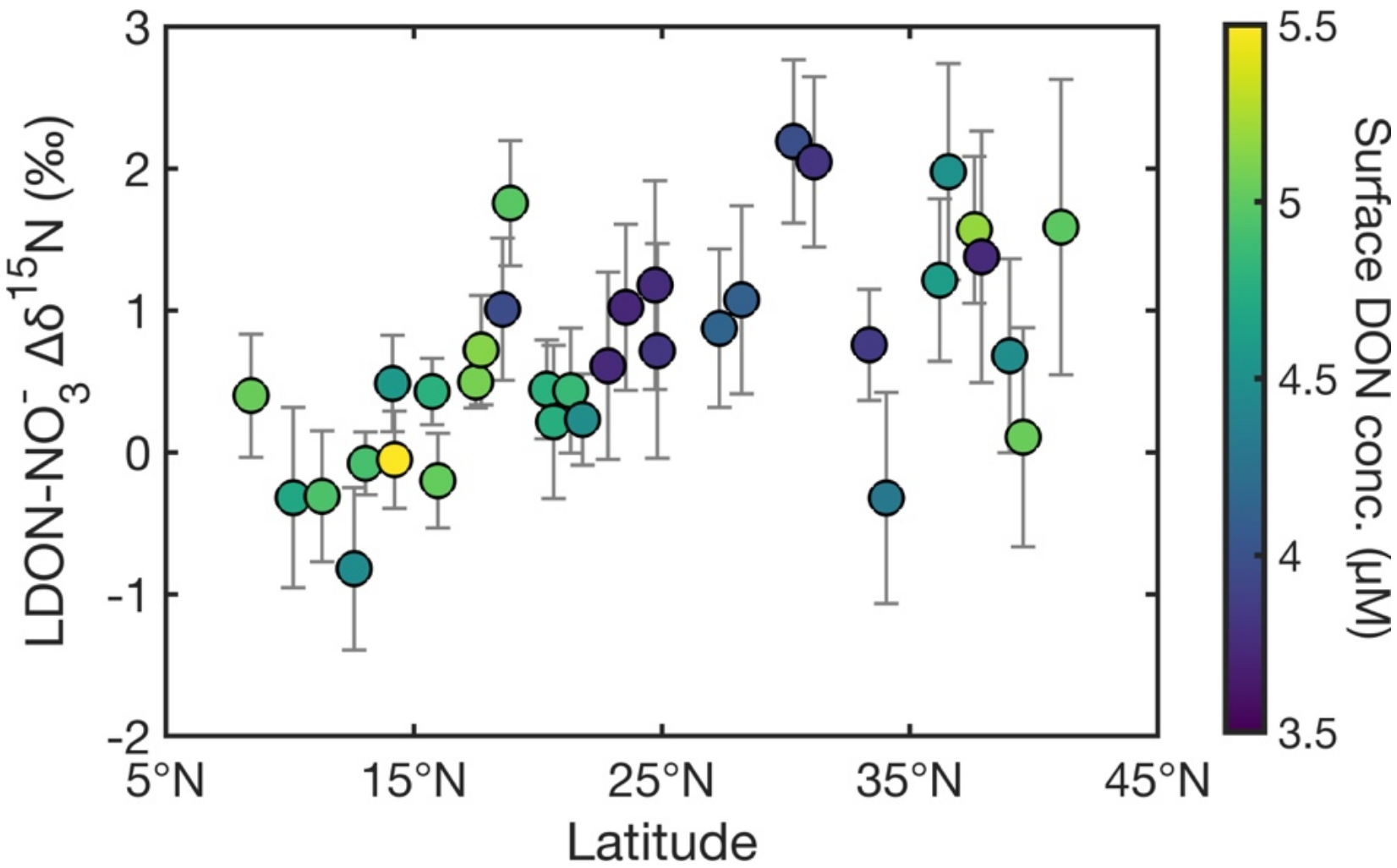

Fig. S8. Latitudinal distributions of LDON-NO_3^- $\Delta\delta^{15}N$ across the Sargasso Sea. Symbols with error bars indicate mean values ± 1SD compiled from the western North Atlantic transects (A20 and A22). Symbols are color-coded by surface DON concentration.

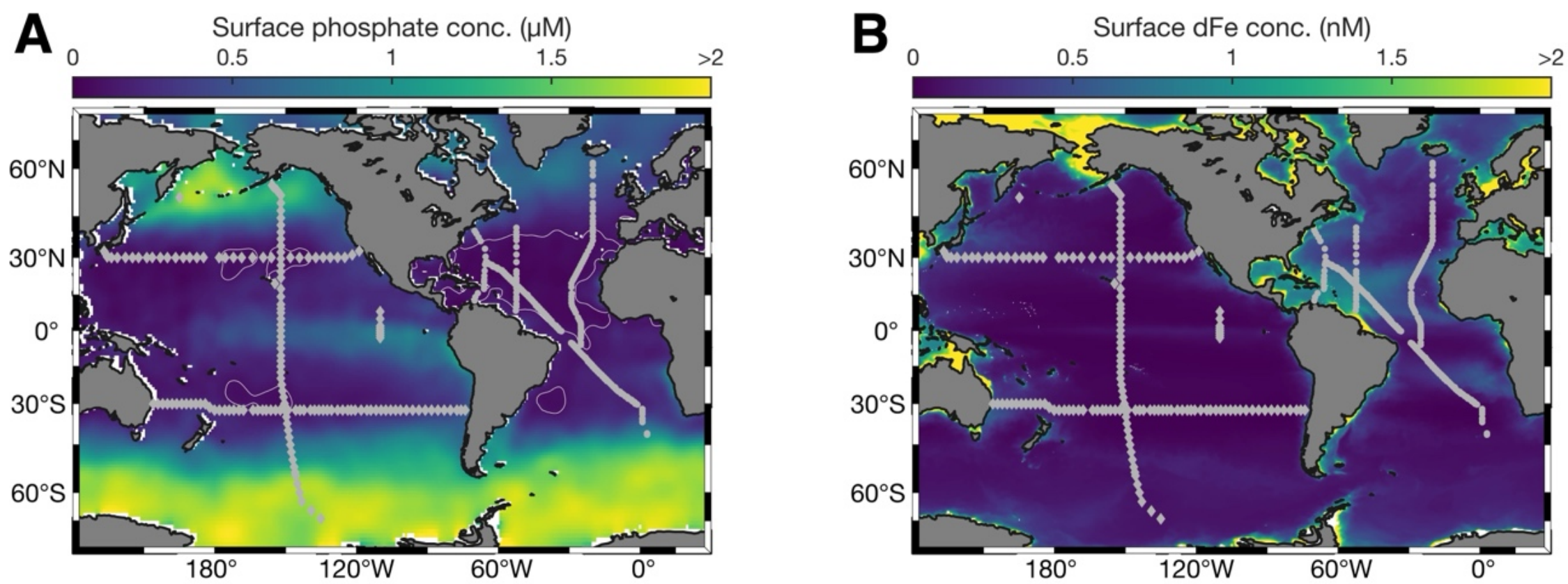

Fig. S9. Annual mean concentrations of (A) phosphate and (B) dissolved iron (dFe) in the surface ocean. The contours in (A) indicate 0.1 µM phosphate. Phosphate data are sourced from the World Ocean Atlas 2018 (12), and dFe data are derived from biogeochemical model outputs (Copernicus Marine Service Information (ref. 10)). Sampling stations for DON measurements are marked with gray symbols (diamonds: Pacific, circles: Atlantic).

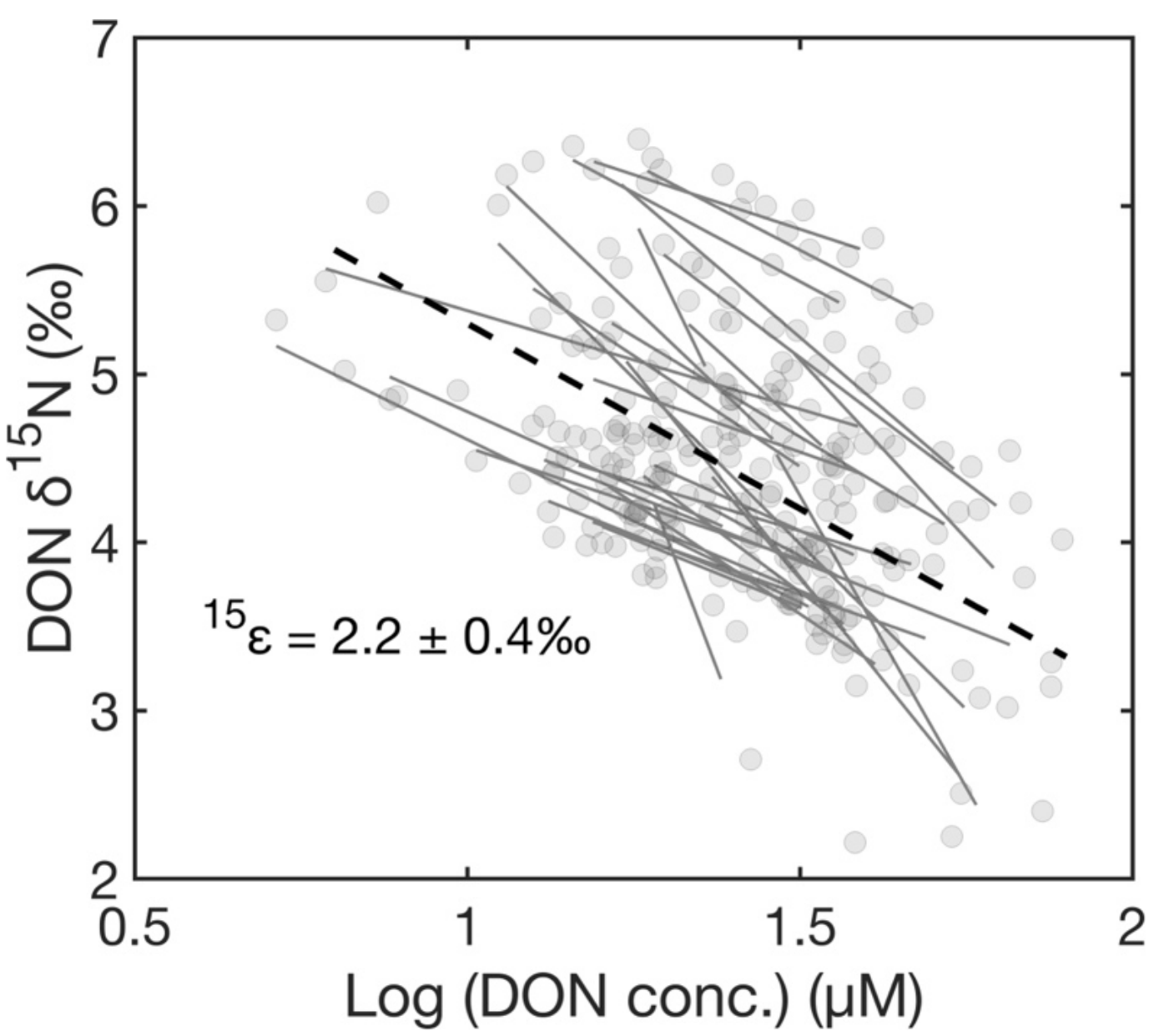

Fig. S10. Estimates of the isotope effect ($^{15}\varepsilon$) for DON decomposition, based on the relationship between the natural logarithm of DON concentration and DON $\delta^{15}N$ from vertical profiles (upper 250 m). Gray dots represent individual data points, gray lines show regressions for each vertical profile, and the dashed black line indicates the weighted mean isotope effect with its 95% confidence interval (2.2 ± 0.4‰).

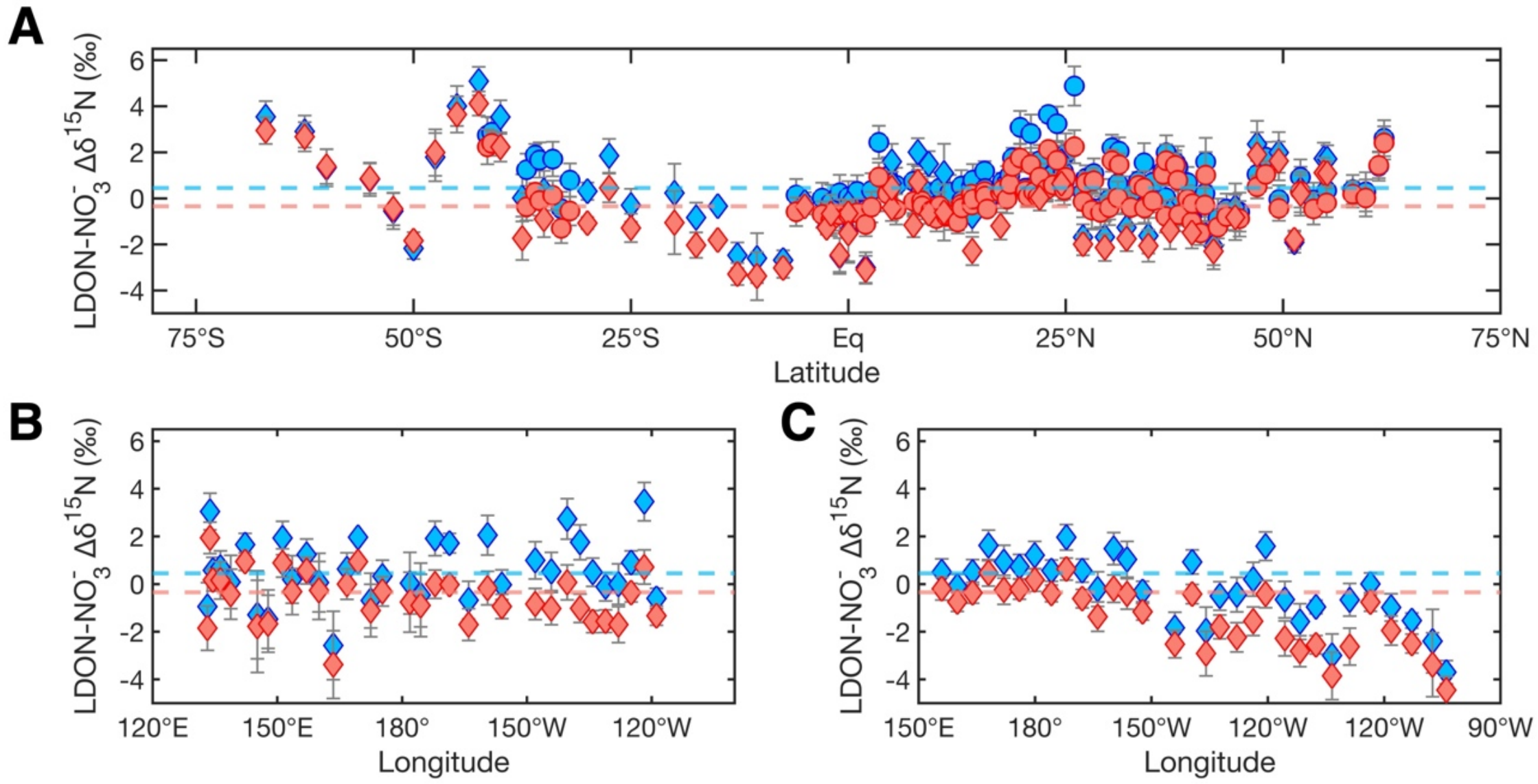

Fig. S11. Sensitivity of LDON-NO_3^- $\Delta\delta^{15}N$ distributions to non-labile endmember selection. (**A**) Meridional and (**B,C**) zonal transects of LDON-NO_3^- $\Delta\delta^{15}N$ calculated using different non-labile DON endmembers. Blue and red symbols represent calculations using Atlantic and Pacific deep DON as the non-labile endmember, respectively. In (A), circles denote Atlantic data and diamonds denote Pacific data. Panels (B) and (C) show zonal transects across the subtropical North Pacific (P02) and South Pacific (P06), respectively. Dashed lines indicate the corresponding mean LDON-NO_3^- $\Delta\delta^{15}N$ values for each endmember scenario.

SI References

1. T. A. Broek *et al.,* Dominant heterocyclic composition of dissolved organic nitrogen in the ocean: A new paradigm for cycling and persistence. *Proc. Natl. Acad. Sci. U.S.A. 120*(49), e2305763120 (2023).
2. R. K. Foreman *et al.,* Improved ultraviolet photo-oxidation system yields estimates for deep-sea dissolved organic nitrogen and phosphorus. *Limnol. Oceanogr. Methods*. 17(4), 277–291 (2019).
3. A. N. Knapp, D. M. Sigman, F. Lipschultz, N isotopic composition of dissolved organic nitrogen and nitrate at the Bermuda Atlantic Time-series study site. *Global. Biogeochem. Cycles.* 19, 1–15 (2005).
4. S. E. Fawcett, B. B. Ward, M. W. Lomas, D. M. Sigman, Vertical decoupling of nitrate assimilation in the Sargasso Sea, *Deep. Sea. Res. Part I. Oceanogr. Res. Pap.* 103, 64–72 (2015).
5. T. Freudenthal *et al.,* Early diagenesis of organic matter from sediments of the eastern subtropical Atlantic: evidence from stable nitrogen and carbon isotopes. *Geochim. Cosmochim. Acta.* 65, 1795–1808 (2001).
6. J. Möbius, Isotope fractionation during nitrogen remineralization (ammonification): Implications for nitrogen isotope biogeochemistry. *Geochim. Cosmochim. Acta.* 105, 422–432 (2013).
7. A. N. Knapp, K. L. Casciotti, M. G. Prokopenko, Dissolved Organic Nitrogen Production and Consumption in Eastern Tropical South Pacific Surface Waters. *Global. Biogeochem. Cycles.* 32, 769–783 (2018).
8. R. Zhang *et al.,* Dissolved Organic Nitrogen cycling in the South China Sea from an isotopic perspective. *Global. Biogeochem. Cy.* 34, (2020).
9. A. Tagliabue *et al.,* A global compilation of dissolved iron measurements: Focus on distributions and processes in the Southern Ocean. *Biogeosciences.* 9, 2333–2349 (2012).
10. Mercator Ocean International, Global Ocean Biogeochemistry Analysis and Forecast (GLOBAL_ANALYSISFORECAST_BGC_001_028). E.U. Copernicus Marine Service Information (CMEMS), Marine Data Store. https://doi.org/10.48670/moi-00015. Accessed March 2024.
11. D. A. Hansell *et al.,* Compilation of dissolved organic matter (DOM) data obtained from global ocean observations from 1994 to 2021. Version 2 (NCEI Accession 0227166) (2021).
12. H. E. Garcia *et al.,* Dissolved inorganic nutrients (phosphate, nitrate and nitrate + nitrite, silicate). *World Ocean Atlas 2018.* 4, 35 (2019).
